# A theoretical framework for brain–computer interfaces: decodability, performance limits, and closed-loop adaptation

**DOI:** 10.3389/fnhum.2026.1964902

**Published:** 2026-09-16

**Authors:** Yunfa Fu, Liu Yan, Xiaogang Chen, Jiahui Pan, Fan Wang, Tianwen Li, Lei Zhao, Xue Yang, Rongzhang Luo, Jiaping Xu, Xinyi Gu

**Affiliations:** 1 Faculty of Information Engineering and Automation, Kunming University of Science and Technology, Kunming, China; 2 Brain Cognition and Brain-Computer Intelligence Integration Group, Kunming University of Science and Technology, Kunming, China; 3 Institute of Biomedical Engineering, Chinese Academy of Medical Sciences, Tianjin, China; 4 School of Artificial Intelligence, South China Normal University, Foshan, China; 5 Faculty of Science, Kunming University of Science and Technology, Kunming, China; 6 Department of Rehabilitation Medicine, The Second Affiliated Hospital of Kunming Medical University, Kunming, China; 7 School of Education, Beijing Institute of Technology, Beijing, China

**Keywords:** BCI theory, brain–computer interface (BCI), information-theoretic constraints, neuroscientific principles, scientific hypotheses, statistical decision theory

## Abstract

Although brain–computer interfaces (BCIs) have made significant advances in brain-signal acquisition and decoding algorithms, the theoretical foundations of BCI remain fragmented, with limited characterization of the decodability, performance bounds, and dynamic constraints of BCI systems. To address this issue, this study proposes a theoretical framework for BCIs from the perspectives of information theory and statistical decision theory. The BCI system is formalized as a closed-loop stochastic process comprising intention generation, neural encoding, signal observation, statistical decoding, and feedback regulation. On this basis, a hierarchical theoretical framework is established that integrates scientific hypotheses, neuroscientific principles, fundamental theorems, and fundamental laws. The fundamental theorems characterize the conditions for intention decodability, the upper bound of observable information, the lower bound of optimal decoding error, and the convergence of closed-loop learning. The fundamental laws reveal the constraints imposed by signal-to-noise ratio, low-dimensional neural representations, irreversible loss of observational information, class-distribution separability, and non-stationary co-adaptation. Building on this, the paper takes a four-class SSVEP-BCI as a theoretical case study, parameterizes and instantiates the aforementioned theorems and laws, verifies the operability and explanatory power of the proposed framework within a concrete paradigm, and translates the theory into actionable design guidelines for BCI systems. To the best of our knowledge, this paper is the first attempt to propose and systematically elaborate a theoretical framework for BCI from a unified theoretical perspective, and it is expected that this work will provide a theoretical foundation for the analysis, design, and optimization of BCI systems.

## 1 Introduction

Brain–computer interfaces (BCIs) aim to achieve direct communication and control between the human brain and external devices by analyzing central neural signals (Wolpaw et al., 2002; Chen et al., 2024; Gao et al., 2025; Fu et al., 2026). Although existing studies have analyzed and designed BCI systems from the perspectives of brain signal acquisition, preprocessing, feature extraction, and translation (Graimann et al., 2010; Allison et al., 2012; Wolpaw and Wolpaw, 2012; Ramsey and Millán, 2020), most of them focus on localized problems or specific task scenarios, and a theoretical framework capable of describing the entire process of intention generation, neural encoding, signal observation, statistical decoding, and feedback regulation is still lacking. A core scientific question that has not been fully answered is: under what conditions can user intention be reliably decoded from limited and noisy brain signals, and what fundamental constraints does this process face theoretically?

Owing to the lack of theoretical support, current BCI research tends to emphasize specific algorithms and task performance optimization, while in-depth theoretical analysis remains insufficient for key issues such as the necessary conditions for intention decodability, information loss during observation, theoretical bounds on optimal decoding performance, and the dynamic evolution laws of the system under closed-loop interaction. This “method-heavy, theory-light” research paradigm limits, to a certain extent, the understanding across different BCI paradigms and also hinders the transition of such systems from laboratory research to stable real-world applications.

In BCI research, the application of an information-theoretic perspective can be traced back to the information transfer rate calculation method proposed by Wolpaw et al. (2002). This method abstracts a BCI system as a discrete memoryless communication channel, taking the total number of target classification classes and the classification accuracy as core parameters to define the amount of information transferable per trial. The single-trial information is then multiplied by the number of trials that can be completed per unit time, ultimately yielding the information transfer rate in bits per minute (Wolpaw et al., 2002). Since its introduction, this calculation method has been used for performance evaluation of various BCI systems. Subsequent studies have extended it in multiple directions: Gage et al. (2006) proposed the concept of information capacity, which quantifies the maximum information rate that the signal itself can provide rather than the empirically measured rate under specific experimental parameters; Kronegg et al. (2005) analyzed the applicability conditions and underlying assumptions of various bit-rate definitions. Furthermore, mutual information has also been used for channel selection and feature extraction optimization in BCIs, and information bottleneck theory has been employed to account for the low-dimensional structure of neural representations (Lan et al., 2006; Buesing and Maass, 2010).

However, the aforementioned studies have mainly focused on performance metrics at the output end of BCI systems or information optimization of specific components, and a theoretical framework spanning intention generation, neural encoding, signal observation, statistical decoding, and feedback regulation has yet to be established. Based on this scientific proposition, this paper proceeds from information theory and statistical decision theory, and unfolds layer by layer following the logic chain of “system formalization and core scientific questions (Q) → scientific hypotheses (H) → neuroscientific principles (P) → fundamental theorems (T) → fundamental laws (L) → design criteria” to construct a BCI theoretical system, as illustrated in Figure 1.

**Figure 1:**
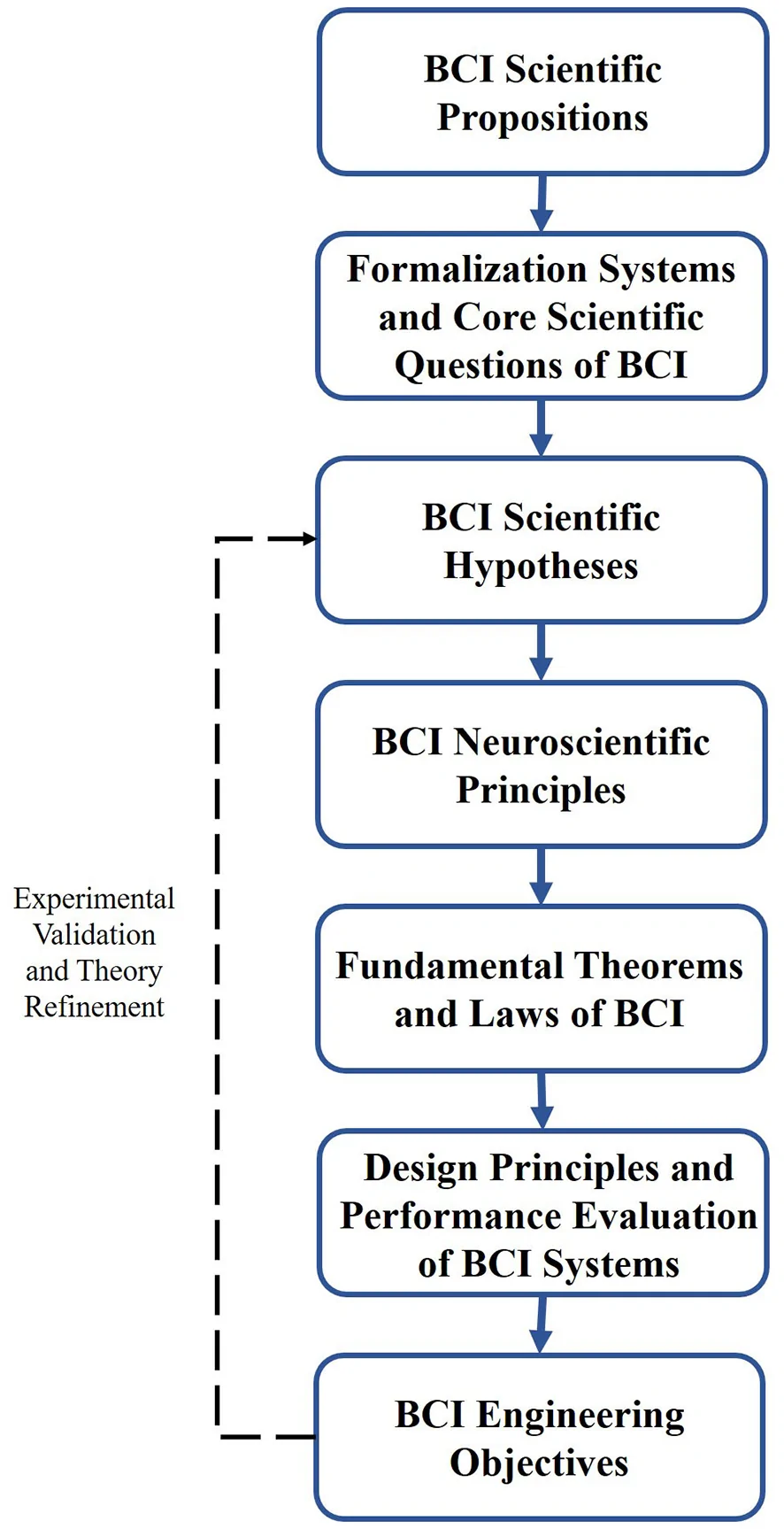
The hierarchical structure and logical chain of the BCI theoretical framework developed in this study.

The logic chain illustrated in Figure 1 forms a complete “theoretical closed-loop structure,” thereby establishing a theoretical framework that describes intention decodability, information transmission constraints, performance limits, and dynamic behavior in BCI systems.

The main contributions of this paper are as follows:

1) The BCI problem is formalized as a closed-loop probabilistic information processing system of “intention generation—neural encoding—signal observation—statistical decoding—feedback regulation,” and a mathematical description framework is established.
2) Core scientific questions and hierarchical scientific hypotheses for BCIs are proposed, and a unified theoretical analysis framework oriented toward closed-loop BCI systems is constructed. Based on classical information theory and statistical decision theory, the necessary condition for intention decodability, information transmission constraints, and theoretical performance bounds are derived and formally characterized for the entire BCI information processing chain.
3) Based on the constructed BCI theoretical framework, BCI system design criteria are proposed, providing a theoretical foundation for the analysis, design, optimization, and long-term deployment of BCI systems.

It should be noted that this study is positioned as a theoretical framework for BCIs and focuses on the analysis of fundamental theoretical issues. Its primary objective is to establish a formal description, formulate scientific hypotheses, and elucidate the fundamental constraints governing BCI systems, rather than to optimize experimental performance for specific task scenarios. Accordingly, the study mainly presents the derivation rationale and theoretical interpretation of the relevant theorems, with the aim of highlighting their implications for the analysis and design of BCI systems. The central contribution of this work is to address the current lack of a unified theoretical framework for analyzing BCI systems, thereby helping to fill an important gap in the theoretical foundations of the BCI field.

## 2 Formalization of the BCI system, core scientific questions, and scientific hypotheses

The essence of BCI is not merely a brain signal classification problem, but a dynamic closed-loop information processing process involving intention generation, neural encoding, signal observation, statistical inference, and feedback regulation. Therefore, this section first constructs a formal model of the BCI system, then presents the core scientific questions of interest in BCI, and further proposes scientific hypotheses that underpin the subsequent theoretical analysis.

### 2.1 BCI system formalization and notational conventions

A BCI system is essentially a dynamic closed-loop probabilistic information processing system driven by user intention, realized through the generation of central neural activity, and achieving communication and control with external devices via observation and inference. Unlike traditional open-loop modeling, in a real BCI system, the decoding result acts on the user through multimodal feedback such as visual, auditory, and tactile modalities, thereby modulating their subsequent mental activity strategies and central neural activity, forming a sustained closed-loop human–machine interaction (Curran and Stokes, 2003; Collura, 2014; Sorrell et al., 2021). To characterize the information flow and stochastic processes in this closed-loop system, all random variables and stochastic processes are defined on the same complete probability space (Ω, F, P), where Ω is the sample space, F is a σ-algebra on Ω, and P is a probability measure. The time index set is taken as the discrete positive integer set t ε N^+^, and the system evolves stepwise in time. This paper introduces the following notational conventions and provides a formal description of the fundamental scientific problems in the BCI system.

1) At time *t*, the user intention is defined as a random variable *I*_*t*_ on the intention space I, $I_{t}\in I$. This variable represents the intention that the BCI system needs to infer, serving as the implicit input of the system. It cannot be directly observed by the device and can only be indirectly inferred through neural activity. Two scenarios, discrete and continuous, are distinguished: under discrete classification tasks, I={1,2,…,C} is a finite set of categories, corresponding to *C* discrete control intentions, with the prior distribution *p*(*I*_*t*_ = *i*) = π_*i*_, satisfying $\overset{C}{\underset{i=1}{\sum }}\pi_{i}=1$; under continuous control tasks, $I\subseteq {\mathbb{R}}^{d}$ is a closed subset of d-dimensional Euclidean space, corresponding to motion trajectories, continuous control quantities, etc., with the prior probability density *p*(*I*_*t*_ ).
2) At time *t*, the true central neural activity is defined as a time-varying stochastic process *A*_*t*_, $A_{t}\in {\mathbb{R}}^{n}$. This variable can be neuronal firing, local field potentials, large-scale neural population activity, etc., and is also a latent variable that cannot be directly observed. The dimension n represents the dimensionality of the latent neural representation space. Given the intention *I*_*t*_ = *i*, the neural activity follows the conditional distribution *p*(*A*_*t*_∣*I*_*t*_ = *i*), which characterizes the neural encoding mechanism of the intention.
3) At time *t*, the brain signal obtained by the measurement device is defined as the observed signal *S*_*t*_, $S_{t}\in {\mathbb{R}}^{m}$. This signal is the result of central neural activity *A*_*t*_ after physiological propagation and sensor acquisition, such as neuronal spikes, ECoG, EEG, or fMRI. The observation dimension *m* reflects the resolving power of the measurement system; typically *m* ≪ *n*, reflecting the incompleteness and dimensionality reduction of the observation process. The observation process is characterized by the conditional distribution *p*(*S*_*t*_∣*A*_*t*_), which corresponds to the complete process of physiological propagation, volume conduction, and sensor acquisition of neural activity.
4) At time *t*, the decoded output intention is defined as the estimate Î_*t*_ of the user intention *I*_*t*_ obtained through a decoding model based on the observed signal *S*_*t*_, ${Î}_{t}\in I$. This variable is essentially the result of statistical inference on the random variable *I*_*t*_.

This paper models the BCI system as a dynamic generation–observation–inference–feedback process with feedback coupling. At time *t*, the evolutionary structure of the system is given by Equation 1, its joint probability distribution is characterized by Equation 2, and the feedback mechanism is formally described by Equations 3, 4.


$$\begin{array}{l} I_{t}\to A_{t}\to S_{t}\to {Î}_{t}\to I_{t+1} \end{array}$$ (1)

$$\begin{array}{l} P\left(I_{t},A_{t},S_{t},{Î}_{t}\right)=P\left(I_{t}\right)P\left(A_{t}|I_{t}\right)P\left(S_{t}|A_{t}\right)P\left({Î}_{t}|S_{t}\right) \end{array}$$ (2)

$$\begin{array}{l} I_{t+1}\sim P\left(I_{t+1}|{Î}_{t},I_{t}\right) \end{array}$$ (3)

$$\begin{array}{l} P\left(A_{t+1}|I_{t+1}\right)\neq P\left(A_{t}|I_{t}\right) \end{array}$$ (4)

Here, *P*(*A*_*t*_∣*I*_*t*_) describes the encoding mapping from intention *I*_*t*_ to central neural activity *A*_*t*_ ; *P*(*S*_*t*_∣*A*_*t*_) characterizes the mechanism by which central neural activity generates the observed signal *S*_*t*_ through physiological propagation and measurement processes; and *P*(Î_*t*_∣*S*_*t*_) represents the decoding process that performs statistical inference of the intention based on the observed signal. Equation 4 further indicates that under the influence of feedback, the statistical properties of the BCI system evolve over time, manifesting as non-stationarity. This structure bears formal similarity to partially observable Markov decision processes (POMDPs) at the level of “latent state–observation–feedback update” (Bellman, 1957; Bäuerle and Jaśkiewicz, 2024); however, this paper does not rely on the standard POMDP framework for derivation, but rather employs it as a conceptual description of the closed-loop stochastic dynamic characteristics.

### 2.2 Core scientific questions of BCI

The core scientific questions of BCI can be summarized as a systematic investigation of the feasibility, conditional constraints, and theoretical limits of decoding user intention, mainly encompassing the following three aspects:

1) Whether a user's specific intention can be effectively inferred from limited and noisy observed brain signals;
2) Under what conditions and constraints user intention is decodable;
3) What theoretical limits and practical performance bounds the decoding capability of a BCI system can achieve.

The above questions constitute the fundamental starting point of the BCI theoretical framework, directly determining the possibility, implementation path, and performance ceiling of decoding user intention from brain signals. Answering these questions not only provides a clear problem orientation for the theoretical modeling of BCI systems, but also lays down the basic premises and constraints for subsequent method design, performance analysis, and system optimization.

### 2.3 BCI scientific hypotheses

This paper proposes four BCI scientific hypotheses to be verified, corresponding respectively to the four levels of encoding, observation, decoding, and feedback: the hypothesis of neural encodability of intention, the hypothesis of observability of intention-related neural activity, the hypothesis of neural decodability of intention, and the hypothesis of feedback modulation of intention neural encoding, as presented in Table 1. Unless otherwise stated, “neural” in this paper refers specifically to the central nervous system (CNS).

**Table 1:** Core scientific hypotheses of BCI.

Hypothesis level	Hypothesis	Statement	Core scientific question
Encoding	H1: Neural Encoding of Intentions Hypothesis	Specific user intentions are assumed to give rise to stable or distinguishable neural representations within the central nervous system.	Can user intentions be represented by central neural activity?
Observation	H2: Observability of Intention-Related CNS Activity Hypothesis	Neural activity associated with specific user intentions is assumed to be partially accessible to measurement systems under appropriate conditions.	Can intention-related central neural activity be observed or measured?
Decoding	H3: Neural Decodability of Intentions Hypothesis	Specific user intentions are assumed to be inferable from observed central neural signals through appropriate decoding processes.	Can user intentions be inferred from central neural signals?
Feedback	H4: Feedback-Modulated Neural Encoding Hypothesis	Decoding outputs and their associated feedback are assumed to influence cognitive states and neural activity, thereby altering the distribution of subsequent neural representations of intentions.	Can feedback modify neural encoding and the separability of intention representations?

These hypotheses are not *a priori* necessarily true; rather, they are empirical premises that require experimental verification under specific tasks, observation conditions, and neural scales. For instance, under certain highly abstract cognitive states or extremely low signal-to-noise ratio conditions, the relevant neural representations may not possess stable observability or statistical separability (Dong et al., 2023; Tai et al., 2024). The core work of this paper is not to prove these hypotheses themselves, but to derive quantitative criteria for BCI system decodability, theoretical performance bounds, and closed-loop evolution laws through information-theoretic and statistical decision tools under the premise that these hypotheses hold. The truth of the hypotheses must be empirically tested through neuroscientific experiments, while the theoretical system constructed in this paper provides quantitative analysis tools for hypothesis testing and boundary determination.

To further clarify the applicable boundaries of the hypotheses and prevent their unrestricted generalization, Table 2 elaborates on the validity conditions and empirical verification approaches for each hypothesis separately.

**Table 2:** Validity conditions and empirical verification routes for each hypothesis.

Hypothesis	Core premise for validity	Scenarios of validity/invalidity	Experimental verification method
H1: Neural Encoding of Intentions Hypothesis	Distinct intentions correspond to stable, reproducible central nervous activity patterns.	Valid: motor execution/imagery, sensory stimulation, basic cognitive tasks with clear brain functional mapping.	Multivariate Pattern Analysis (MVPA)is employed to test statistical differences in neural activity across distinct intentions, and encoding stability is verified through the reproducibility of neural representations within trials and across sessions (e.g., representational similarity analysis, RSA).
H2: Observability of Intention-Related CNS Activity Hypothesis	Validity depends on spatiotemporal resolution of recording modalities, depth & distribution of neural sources, and mixing degree of physiological propagation.	Strong validity: invasive spike recording, ECoG with high fidelity for cortical activity.	Source imaging and task-state signal-to-noise ratio analysis are used to quantify the acquirability of intention-related neural components; differences in intention-related mutual information between invasive (Spike/ECoG) and non-invasive (EEG/fNIRS) modalities are compared to verify the correspondence between observational fidelity and modal spatial resolution.
H3: Neural Decodability of Intentions Hypothesis	Prerequisites: H1 and H2 hold; observed signal distributions of different intentions are statistically separable.	Decoding fails when there are excessive intent categories, extremely low SNR, or heavily overlapped neural representations (mutual information between intent and signals approaches zero).	Permutation testing is adopted to determine whether decoding accuracy is significantly above the random-guessing baseline, and mutual information estimation is combined to verify the presence of non-zero intention information in the observed signals.
H4: Feedback-Modulated Neural Encoding Hypothesis	Subjects possess intact neural plasticity and cognitive regulation capacity.	Strong validity: healthy subjects and most rehabilitative patients (closed-loop feedback adapts neural representations).	A longitudinal closed-loop training controlled experiment is conducted to validate the regulatory effect of feedback on neural encoding, assessed through changes in neural representation separability (e.g., Fisher discriminant ratio, between-class distance) before and after feedback, as well as learning curves of decoding performance.

The above scientific hypotheses elevate BCI from a purely engineering implementation problem to a theoretical framework consistent with neuroscientific. Their core role is to explain why a BCI system is fundamentally feasible and why it is constrained, and to provide the basic premises for the subsequent theoretical derivations.

Structurally, these hypotheses together form a hierarchical “encoding–observation–decoding–feedback” closed-loop system, corresponding to the processes of information generation, observation, inference, and regulation: user intention drives neural encoding, observable signals are formed through neural activity, the observed signals are decoded to estimate the intention, and the decoding result further acts on the user through feedback, modulating their subsequent cognitive state and neural activity, thereby forming a continuously evolving closed-loop human–machine interaction.

It should be emphasized that in the decoding hypothesis (H3), “neural decoding” is not equivalent to necessarily adopting a biologically plausible model; rather, it broadly refers to the process of statistically inferring the user's intention from central neural signals.

Furthermore, the feedback hypothesis (H4) introduces temporal dynamics and adaptive mechanisms, whose essence lies in characterizing the modulatory effect of feedback on the neural encoding process. That is, neural representations and their statistical distributions can change during interaction, thereby endowing the BCI system with characteristics of non-stationarity and learnability.

## 3 Neuroscientific principles underpinning BCI theory

In Section 2, this paper proposed the formal model, core scientific questions, and four scientific hypotheses (H1–H4) of the BCI system. Building upon this, the present section further proposes four neuroscientific principles (P1–P4) corresponding to the above hypotheses, derived from neuroscientific and information processing laws, to characterize the intrinsic mechanisms of intention encoding, signal observation, intention decoding, and closed-loop feedback regulation in the BCI system. These principles are not intended to hold as rigorous mathematical propositions, but rather serve as abstract generalizations of a large body of neuroscientific empirical findings and BCI empirical regularities. Their role is to provide the mechanistic explanations on which the subsequent theoretical analysis relies.

The BCI neuroscientific principles possess cross-task and cross-modality generality, and serve as a bridge connecting the scientific hypotheses and the fundamental theorems in the theoretical system: on the one hand, they provide a neural mechanism basis for the subsequent theoretical results; on the other hand, they correspond one-to-one with the probabilistic formalized model defined in Section 2, providing explicit mathematical premise constraints for theorem derivation, and offering a unified explanatory framework for the performance limitations and feasibility of BCI systems.

### 3.1 P1: principle of distributed neural encoding of intention

This principle states that user intention is encoded in a distributed manner in the central nervous system, i.e., neural representations are formed through the coordinated activity of multiple neurons or multiple brain regions, rather than the independent expression of a local single neural unit (Yuste et al., 2024). At the observation level, this distributed encoding manifests as extractable neural features.

This principle corresponds to the encoding layer (*I*→*A*). From a probability distribution perspective, the neural encoding distribution of intention *p*(*A*_*t*_∣*I*_*t*_) is the joint distribution of the activities of multiple brain regions and neural subpopulations, satisfying the statistical properties of distributed representations, and the activity of a single neural unit cannot fully represent the intention information. Formally, intention-related neural activity can be expressed as the superposition of activities from multiple brain regions, as shown in Equation 5.


$$\begin{array}{l} A_{t}=\overset{K}{\underset{k=1}{\sum }}a_{k}\left(t\right) \end{array}$$ (5)

where *a*_*k*_(*t*) denotes the activity of the *k*-th brain region or neural subpopulation.

### 3.2 P2: principle of multi-source aliasing and incomplete observation of intention-related neural signals

This principle states that the observed signal is the result of mixing multi-source activities, including multiple neural sources as well as confounding components such as non-neural physiological sources (e.g., electromyographic and electrooculographic signals), environmental interference, and stimulus artifacts (Khan et al., 2024). Some of these confounding components may be indirectly correlated with the task intention, disrupting the pure Markov chain structure of “intention–neural–observation” and serving as a significant source of interference in signal analysis for real BCI systems. Furthermore, due to the limited number of sensors and the volume conduction effect, the observation process provides an incomplete sampling of the true neural activity, thereby leading to irreversible information loss (Sun et al., 2024).

This principle corresponds to the observation layer (*A*→*S*), and is fully consistent with the observation channel model defined in Section 2.1: the observation process is equivalent to a noisy non-linear mapping; under linear approximation, it manifests as a linear mixture of multi-source signals, with the noise term statistically independent of the neural activity; at the same time, the observation dimension is much lower than the neural state dimension, the observation mapping is a non-bijective mapping, and information loss inherently exists. Its formal expression is given by Equation 6, or linearly approximated as Equation 7, and satisfies Equation 8.


$$\begin{array}{l} S_{t}=H\left(A_{t}\right)+\varepsilon_{t} \end{array}$$ (6)

$$\begin{array}{l} S_{t}=WA_{t}+\varepsilon_{t} \end{array}$$ (7)

$$\begin{array}{l} \dim\left(S\right)\ll \dim\left(A\right) \end{array}$$ (8)

### 3.3 P3: principle of low-dimensional task-relevant neural representation of intention

This principle states that although neural activity as a whole possesses high-dimensional characteristics, the information related to a specific intention or task is typically embedded in a low-dimensional subspace (Langdon et al., 2023). This low-dimensional structure provides the structural basis for the decodability of intention.

This principle corresponds to the decoding layer ($S\to \hat{I}$). From a mathematical structure perspective, the intention-related neural component *A*_*I*_(*t*) is embedded in a low-dimensional subspace of the high-dimensional neural activity, and its intrinsic dimensionality is much smaller than the full neural space dimension *n*; this low-dimensional subspace carries the primary intention-discriminative information, while the remaining dimensions mostly consist of task-irrelevant noise and spontaneous neural activity. Formally, the neural activity can be decomposed as in Equation 9, satisfying Equation 10.


$$\begin{array}{l} A_{t}=A_{I}\left(t\right)+A_{noise}\left(t\right) \end{array}$$ (9)

$$\begin{array}{l} \dim\left(A_{I}\right)\ll \dim\left(A_{t}\right) \end{array}$$ (10)

where *A*_*I*_(*t*) denotes the intention-related component, and *A*_*noise*_(*t*) denotes irrelevant noise.

### 3.4 P4: principle of closed-loop adaptivity and plasticity of intention neural representation

This principle states that under the influence of closed-loop feedback, the neural representation of user intention undergoes dynamic regulation and optimization over time, manifested as the non-stationarity of the neural encoding distribution (Ramsey and Millán, 2020; Jin et al., 2024). This adaptivity originates from learning processes and neural plasticity, causing the BCI system to evolve continuously during interaction.

This principle corresponds to the feedback layer ($\hat{I}\to I\to A$), and is consistent with the closed-loop feedback dynamics model: feedback drives the dynamic updating of the neural encoding distribution, i.e., *p*(*A*_*t*+1_∣*I*_*t*+1_ = *i*)≠*p*(*A*_*t*_∣*I*_*t*_ = *i*), which is also the core source of statistical non-stationarity in BCI systems. It can be formally expressed as Equation 11.


$$\begin{array}{l} A_{t+1}=F\left(A_{t},feedback\right) \end{array}$$ (11)

In summary, P1–P4 characterize the fundamental mechanisms of the BCI system from four aspects—neural representation, signal observation, information structure, and closed-loop adaptivity—and provide a mechanistic foundation for the derivation of the subsequent theorems.

## 4 BCI fundamental theorems

This section further formalizes the BCI neuroscientific principles characterized by P1–P4 above and proposes a set of BCI theorems, Theorems 1–4 (T1–T4). From the perspectives of information theory and statistical decision theory, these theorems rigorously characterize the decodability condition, the upper bound of information transfer, the optimal performance bound, and the closed-loop evolution law of the BCI system, elevating the qualitative principles at the neural mechanism level into quantifiable and derivable mathematical conclusions within the modeling framework.

### 4.1 T1: intention decodability theorem

**1) Assumptions**

(1) The intention I is a discrete random variable taking values in a finite category space I={1,2,…,C}, with prior distribution *p*(*I* = *i*) = π_*i*_.
(2) The observed signal S and the intention I are defined on the same probability space, with joint distribution *p*(*I, S* ).
(3) The decoding task adopts the 0–1 loss, and the baseline error rate of random guessing is *P*_*chance*_ = 1−*max*_π_*i*_*iεJ*_.
(4) The sufficiency conclusion in this section holds strictly only under uniform class priors (π_*i*_ = 1/*C*, ∀*i*); when the priors are imbalanced, only necessity always holds, and sufficiency requires additional constraints.

**2) Theorem statement**

If the observed signal S contains non-zero mutual information about the intention I, i.e., satisfying Equation 12,


$$\begin{array}{l} MI\left(I;S\right)>0 \end{array}$$ (12)

then, in a statistical sense, the intention is decodable. Specifically, there exists a mapping (decoder), as in Equation 13,


$$\begin{array}{l} f:S\to Î \end{array}$$ (13)

such that its decoding accuracy is strictly better than the chance level, as shown in Equation 14.


$$\begin{array}{l} P\left(Î=I\right)>P_{chance} \end{array}$$ (14)

Here, *MI*(*I*; *S*) is the mutual information between the intention and the signal, characterizing the amount of information available for decoding. Mutual information is a fundamental quantity in information theory that measures the statistical dependence between random variables (Shannon, 1948; Cover and Thomas, 1991).

**3) Proof**

From the definition of mutual information *MI*(*I*; *S*) = *H*(*I*)−*H*(*I*∣*S*) and the condition *MI*(*I*; *S*) > 0, we obtain *H*(*I*∣*S*) < *H*(*I*), meaning that the observed signal can reduce the uncertainty of the intention. According to Fano's inequality, for any decoder **f**, its classification error rate *P*_*error*_ = *P*(*f*(*S*)≠*I*) satisfies: *H*(*I*∣*S*) ≤ *H*_2_(*P*_*error*_)+*P*_*error*_*log*_2_(*C*−1), where *H*_2_(·) is the binary Shannon entropy function. When *MI*(*I*; *S*) > 0, we have *H*(*I*∣*S*) < *H*(*I*). Substituting into Fano's inequality allows the derivation that the theoretical lower bound of the error rate is strictly smaller than the random guessing error rate *P*_*chance*_. Consider the Bayes optimal decoder: $f^{*}\left(s\right)={\arg max}_{i\varepsilon j}p\left(I=i|S=s\right)$, which achieves the minimum error rate (Bayes error) among all measurable mappings. Since the lower bound of the error rate is better than random guessing, the error rate of the optimal decoder must satisfy *Error*_*Bayes*_<*P*_*chance*_, i.e., there exists a decoder whose performance is strictly better than random guessing.

Based on the above proof, explicit quantitative constraints on decoding performance can be further derived. From Fano's inequality and the upper bound property of the binary entropy *H*_2_(*P*_*e*_) ≤ 1, a universal lower bound for the classification error rate of any decoder is obtained, as in Equation 15 (Cover and Thomas, 2006).


$$\begin{array}{l} P_{e}\geq \frac{H\left(I\right)-MI\left(I;S\right)-1}{\log_{2}\left(C-1\right)} \end{array}$$ (15)

The corresponding theoretical upper bound for classification accuracy is shown in Equation 16.


$$\begin{array}{l} P_{acc}\leq 1-\frac{H\left(I\right)-MI\left(I;S\right)-1}{\log_{2}\left(C-1\right)} \end{array}$$ (16)

This formula establishes a direct quantitative correspondence between the mutual information value and the upper bound of decoding performance: the larger the mutual information, the higher the theoretical upper limit of accuracy; when the mutual information approaches zero, the accuracy upper bound will converge to the chance level.

This theorem provides the weakest necessary condition for decodability, i.e., it only proves the existence of at least one decoder that is statistically strictly better than random guessing. When the mutual information value is extremely small, the performance improvement may be negligible and lack practical engineering application value. Therefore, this theorem is primarily used for feasibility assessment at the theoretical level and cannot directly serve as a design basis for practical systems; practically useful decoding performance requires the mutual information to reach a higher quantitative threshold.

**4) Extension of decodability to multiple scenarios**

The above theorems and quantitative bounds are presented based on the standard discrete classification scenario. For different types of BCI tasks, corresponding chance-level baselines and decodability criteria should be adopted. For class-imbalanced discrete classification scenarios, when the prior distribution of intention categories is imbalanced, the chance level is no longer the uniform random guess 1/*C*, but the accuracy of the optimal constant classifier (which always predicts the class with the maximum prior probability), as shown in Equation 17.


$$\begin{array}{l} P_{acc,\;chance}={max\pi_{i}}_{i\varepsilon J} \end{array}$$ (17)

The corresponding decodability criterion is that the decoding accuracy is strictly greater than the accuracy of this optimal constant classifier. The more imbalanced the prior distribution, the lower the intention entropy *H*(*I*), and the correspondingly lower the mutual information threshold required to achieve the same accuracy.

For continuous control (regression) scenarios, such as motor trajectory decoding, continuous parameter adjustment, and other regression-type BCI tasks, the framework can be extended based on rate-distortion theory:

Define the distortion measure $d\left(i,\hat{i}\right)=||i-\hat{i}{||}^{2}$ (mean squared error). The rate-distortion function *R*(*D*) represents the minimum information rate required under the constraint that the average distortion does not exceed D. The chance level is defined as the mean squared error of a null model (predicting using only the mean of the intention prior distribution), as shown in Equation 18.


$$\begin{array}{l} MSE_{chance}=Var\left(I\right)=E\left[{\left(I-E\left[I\right]\right)}^{2}\right] \end{array}$$ (18)

If *MI*(*I*; *S*) > *R*(*D*), then there exists a decoder *f* such that the decoding mean squared error satisfies $E\left[d\left(I,\hat{I}\right)\right]\leq D<MSE_{chance}$, i.e., the continuous intention can be decoded with a specified precision.

For sequential decoding scenarios, such as sequential decision-making and continuous time-series control BCIs, the mutual information rate per unit time is adopted as the core metric, and the chance baseline is the first-order Markov prediction error relying solely on historical intentions. The decodability criterion is: the time-series mutual information rate provided by the observed signal is greater than zero, and the decoded sequence prediction error is significantly lower than the prediction error based solely on the intention temporal prior.

**5) Decoder learnability under finite samples**

The preceding conclusions are all based on the ideal premise that the joint distribution of the signal is known, proving the theoretical existence of an optimal decoder. However, in real BCI systems, the signal distribution is unknown, and the decoder must be trained from limited, noisy samples; even if an optimal decoder exists in theory, a practical model that outperforms random chance cannot be learned if the sample size is insufficient (Sussillo et al., 2016).

From the perspective of statistical learning theory, let the VC dimension of the hypothesis space to which the decoder belongs be d. To ensure that the generalization error of the trained decoder differs from the optimal Bayes error by no more than ε, with a confidence level of at least 1-δ, the required number of training samples N satisfies Equation 19.


$$\begin{array}{l} N\geq \frac{1}{\varepsilon^{2}}\cdot \Theta \left(d+\log\frac{1}{\delta }\right) \end{array}$$ (19)

This relationship indicates that the higher the model complexity (i.e., the larger the VC dimension), or the higher the required generalization accuracy (i.e., the smaller the allowed error ε), the larger the number of training samples required. Therefore, there exists a constraint relationship, determined by the data scale, between the theoretical expressive capacity of the decoding model and its practical learnability.

For BCI systems, the high dimensionality, low signal-to-noise ratio, inter-individual variability, and non-stationarity of neural data significantly increase the actual sample size required (Sliwowski et al., 2023). In high-noise environments, stable extraction of effective intention information is difficult; differences in neural representations across individuals lead to difficulties in cross-user generalization; and changes in neural states over time cause distribution drift, reducing the effectiveness of static training data. The data scale required for real BCI systems to achieve theoretical performance is usually higher than the ideal lower bound given by statistical learning theory. Therefore, BCI systems have two levels of performance bounds: the theoretical decodability bound determined by the neural information transmission capacity, and the engineering learnability bound determined by the sample size, model complexity, and data stability. Only when both information observability and finite-sample learning constraints are satisfied can theoretical decodability be translated into practical application performance.

**6) Theoretical significance**

This theorem system progressively tightens the constraints on BCI decodability—from the weakest existence condition and quantitative performance bounds, to multi-task scenario adaptation and finite-sample learnability—thereby clarifying the fundamental prerequisites for neural information decodability at the theoretical level and providing criteria for practical system design at the engineering level. Its core significance lies in the following: it establishes that non-zero mutual information is only a necessary but not sufficient condition for decodability, thereby preventing the misinterpretation of theoretical information existence as practical application performance; it unifies the decodability analysis framework for multiple types of BCI systems by extending to diverse task scenarios such as class-imbalanced classification, continuous control, and temporal sequences; and it distinguishes between the existence of a theoretical decoder and engineering learnability under finite-sample conditions, providing guidance for data collection scale, model complexity selection, and practical system deployment.

### 4.2 T2: observation information upper bound theorem

1**) Assumptions**

This theorem is proposed for the ideal, artifact-free baseline observation scenario, which satisfies the following premises:

(1) The random variables *I, A, S* form a Markov chain *I*→*A*→*S*, i.e., *S* is conditionally independent of *I* given *A*; this assumption by default excludes non-ideal factors such as non-neural confounding signals directly correlated with the intention and stimulus-locked artifacts.
(2) The observation model satisfies *S* = *H*(*A*)+ε, where the noise ε is statistically independent of both *I* and *A*.

**2) Theorem statement**

The amount of intention information contained in the observed signal does not exceed the amount of intention information contained in the true neural activity, as expressed in Equation 20.


$$\begin{array}{l} MI\left(I;S\right)\leq MI\left(I;A\right) \end{array}$$ (20)

Equality holds if and only if the observed signal retains all intention-related information contained in the neural activity, which is equivalent to S being a sufficient statistic of A with respect to I. This condition is weaker than requiring the observation mapping to be fully invertible and noise-free; even if the observation process discards neural activity information unrelated to intention, the equality still holds as long as all intention-related information is preserved. A bijective observation mapping with zero noise is merely a sufficient condition for equality, not a necessary one.

**3) Proof**

Given the premise that *I*→*A*→*S* is a Markov chain, according to the data processing inequality in information theory: for any Markov chain *X*→*Y*→*Z*, it always holds that *MI*(*X*; *Z*) ≤ *MI*(*X*; *Y*). Substituting *X* = *I, Y* = *A, Z* = *S* into the data processing inequality directly yields *MI*(*I*; *S*) ≤ *MI*(*I*; *A* ).

Equality holds if and only if there is no information loss in the data processing procedure, i.e., the mapping *A*↦*S* is an invertible transformation without additional noise, corresponding to a completely faithful observation process. The term “no information loss” here specifically denotes the lossless retention of intention-related information, rather than requiring the observed signal to perfectly reconstruct the neural activity A. In essence, this theorem originates from P2: the principle of multi-source aliasing and incomplete observation.

The observation process can only preserve or lose information, never create it, which directly delimits that the upper bound of BCI performance originates from the neural signal itself. The information loss in the observation process can be quantitatively decomposed into two components, as shown in Equation 21. It should be noted that this decomposition is a conceptual one rather than a rigorous mathematical identity; the two types of loss are coupled in practical systems and cannot always be independently and precisely measured. The decomposition is used here to clarify the physical sources of information loss and to provide theoretical targets for optimization directions.


$$\begin{array}{l} I_{loss}=MI\left(I;A\right)-MI\left(I;S\right)=I_{mix}+I_{noise} \end{array}$$ (21)

where *I*_*mix*_ is the irreversible information loss caused by neural source aliasing and dimension compression, determined by the volume conduction effect and the insufficient number of observation channels; *I*_*noise*_ is the information loss introduced by observation noise, determined by the signal-to-noise ratio level. This decomposition provides clear information-theoretic targets for the optimization of BCI observation systems: improving spatial resolution can reduce *I*_*mix*_, and improving signal quality can reduce *I*_*noise*_.

**4) Extension to non-ideal observation scenarios: modeling confounding variables**

In real BCI systems, the observed signal often contains various non-neural components and does not fully satisfy the ideal Markov chain assumption (Cohen, 2017). Based on the relationship between the confounding variables and the intention, two types of scenarios can be distinguished and modeled separately.

In the scenario with independent confounding variables, if the observed signal contains confounding variables C that are independent of the intention (such as ambient electromagnetic noise, spontaneous physiological artifacts, random electromyographic interference), the observation model is modified as Equation 22.


$$\begin{array}{l} S=H\left(A\right)+C+\varepsilon \end{array}$$ (22)

where C is statistically independent of I and A. In this case, the Markov chain structure *I*→*A*→*S* still holds, and the information upper bound constraint of the original theorem remains valid; the effect of the confounding variable is equivalent to raising the noise level, further reducing the actual mutual information. The larger the variance of the confounding variable, the more severe the information loss.

In the scenario with correlated confounding variables, if the observed signal contains non-neural confounding variables C that are directly associated with the intention (such as stimulus-locked artifacts from the stimulation electrodes in SSVEP paradigms, task-related electromyographic co-activation accompanying motor imagery, systematic eye movements concomitant with cognitive tasks, and environmental interference phase-locked to the task), the intention can influence the observed signal through pathways other than neural activity, and the original pure Markov chain structure *I*→*A*→*S* no longer holds. Under this scenario, the total intention-related mutual information in the observed signal comprises two parts, as shown in Equation 23. It should be noted that this decomposition is a conceptual one; in practical observations, neural sources and confounding sources are often intermixed at the signal level, making it difficult to strictly separate and independently quantify them. The distinction made here serves to explain the origins of inflated performance and to provide controls for experimental design.


$$\begin{array}{l} MI\left(I;S\right)=MI{\left(I;S\right)}_{neural}+MI{\left(I;S\right)}_{confound} \end{array}$$ (23)

where the neural-source component *MI*(*I*; *S*)_*neural*_ still satisfies the upper bound constraint of the original theorem, i.e., *MI*(*I*; *S*)_*neural*_ ≤ *MI*(*I*; *A*); whereas the confounding-source component *MI*(*I*; *S*)_*confound*_ originates from spurious correlations through non-neural pathways and does not correspond to genuine neural intention encoding.

In this case, directly using the total mutual information to evaluate decoding capability would overestimate the true neural decoding performance; part of the “higher-than-expected decoding accuracy” observed in actual BCI research comes from such spurious correlations introduced by confounding variables, rather than genuine neural intention decoding.

**5) Theoretical significance**

This theorem clarifies the fundamental constraint of information transfer in the BCI observation stage: the observation process can only preserve or lose neural information, never create new intention-related neural information, and the performance upper bound of a BCI system is fundamentally determined by the amount of intention information present in the neural encoding stage.

Its core value lies not in proposing entirely new information-theoretic conclusions, but in operationalizing the general data processing inequality into a BCI-specific cascaded channel analysis tool, which, through the two-dimensional decomposition of information loss, provides a theoretical explanation for performance differences across observation modalities.

For the confounding variable scenarios in real BCI systems, the theorem's conclusions can be correspondingly extended and revised: the ideal baseline upper bound holds only for information of neural origin, and the spurious correlations introduced by non-neural confounds can lead to spuriously inflated performance, which is a bias that needs to be carefully avoided in BCI experimental design and result interpretation.

### 4.3 T3: statistical optimal error lower bound theorem for BCI intention decoding

1**) Assumptions**

(1) The joint distribution *p*(*I, S*) of the intention *I* and the observed signal *S* is known and fixed.
(2) The decoding task adopts the 0–1 loss, and the decoder is any measurable mapping f:S→ I.

**2) Theorem statement**

For any intention decoder based on the observed signal *S*, its error satisfies Equation 24.


$$\begin{array}{l} Error_{dec}\geq Error_{Bayes} \end{array}$$ (24)

Here, *Error*_*dec*_ denotes the error of a specific intention decoder, i.e., the overall probability of error ℙ (classification error rate) when decoding the intention based on the observed signal *S*, as shown in Equation 25; *Error*_*Bayes*_ denotes the Bayes error, i.e., the average error rate achieved under the maximum a posteriori (MAP) optimal decision strategy, characterizing the minimum possible error (theoretical lower bound) that any decoder can achieve given the signal distribution (Bayes, 1763; Laplace, 1820; Cover and Thomas, 1991; Curran and Stokes, 2003; Bishop and Nasrabadi, 2006), as shown in Equation 26.


$$\begin{array}{l} Error_{dec}=P\left(Î\neq I\right) \end{array}$$ (25)

$$\begin{array}{l} Error_{Bayes}=1-E_{S}\left[\underset{I}{\max}P\left(I|S\right)\right] \end{array}$$ (26)

**3) Proof**

For any fixed observation value s, the conditional error probability corresponding to the decoder output $1-p\left(I=\hat{i}|S=s\right)$. To minimize this conditional error probability, one must select the category that maximizes the posterior probability, i.e., ${\hat{i}}^{*}={\arg\max}_{i}p\left(I=i|S=s\right)$, at which point the conditional error probability achieves its minimum value 1 − max_*i*_*p*(*I* = *i*∣*S* = *s*). Taking the expectation over all observation values yields the overall minimum error rate, as shown in Equation 27.


$$\begin{array}{l} Error_{Bayes}=E_{S}\left[1-\max_{i\varepsilon J}p\left(I=i|S\right)\right]\\ \;=1-E_{S}\left[\max_{i\varepsilon J}p\left(I=i|S\right)\right] \end{array}$$ (27)

Since this error is the expectation of the minimum error rate at each observation point, the error rate of any other decoder cannot be lower than this value. That is, for any *f*, *Error*_*dec*_≥*Error*_*Bayes*_.

**4) Quantitative relationship between information-theoretic bounds and performance metrics**

Equation 15 directly provides the lower bound of the classification error rate given a certain mutual information, which is also the upper bound of the classification accuracy, thus realizing a quantitative conversion between information-theoretic bounds and engineering performance metrics. On this basis, the theoretical constraint on the information transfer rate (ITR) can be further derived: the standard definition of the information transfer rate for a BCI system is given by Equation 28.


$$\begin{array}{l} ITR=\frac{1}{T}\left[\log_{2}C+P\log_{2}P+\left(1-P\right)\log_{2}\frac{1-P}{C-1}\right] \end{array}$$ (28)

where *T* is the duration of a single trial, and *P* is the classification accuracy. By combining the constraint relationship between mutual information and error rate, the information-theoretic upper bound of the ITR can be derived, as shown in Equation 29.


$$\begin{array}{l} ITR\leq \frac{MI\left(I;S\right)}{T} \end{array}$$ (29)

That is, the information transfer rate per unit time cannot exceed the intention-related mutual information carried by the observed signal per unit time, providing a theoretical ceiling for the communication efficiency of BCI systems.

**5) Key factors influencing the Bayes error in BCI scenarios**

The Bayes error is fundamentally determined solely by the degree of overlap of the class-conditional distributions in the observation space. In the context of the BCI-specific observation chain, its magnitude is systematically regulated by multiple categories of core factors, each corresponding to explicit quantitative constraints. Under a typical observation model with additive Gaussian noise, if the observation distributions corresponding to two intention classes are Gaussian with means μ_1_, μ_2_ and covariance matrix Σ, then the Bayes error and the discriminability index *d*′ satisfy Equation 30.


$$\begin{array}{l} Error_{Bayes}=\Phi \left(-\frac{d^{\prime }}{2}\right),\;d^{\prime }=\sqrt{{\left(\mu_{1}-\mu_{2}\right)}^{T}\Sigma^{-1}\left(\mu_{1}-\mu_{2}\right)} \end{array}$$ (30)

where Φ(·) is the cumulative distribution function of the standard normal distribution. The signal-to-noise ratio (SNR) is positively correlated with *d*′: the higher the SNR, the lower the overlap of class distributions and the smaller the Bayes error. This relationship directly binds the signal quality index at the hardware level to the theoretical performance lower bound.

The observation dimension m (number of electrode channels) and the spatial distribution also play a critical role, as they determine the severity of source aliasing: the lower the observation dimension and the poorer the spatial resolution, the more severe the overlap of the projections of high-dimensional neural sources in the low-dimensional observation space, leading to a higher overlap of class-conditional distributions and a larger Bayes error. Increasing the number of effective observation channels can reduce the degree of source aliasing and decrease the Bayes error; however, limited by the volume conduction effect, the performance gain exhibits a trend of diminishing marginal returns, which is fully consistent with the L4 Law of Observation Dimensionality Constraint.

For the conditional distributions of any two classes of intention, the Bhattacharyya distance can simultaneously provide both upper and lower bounds on the Bayes error, thereby forming a two-sided constraint on the optimal classification error, as shown in Equation 31 for the case of uniform binary class priors.


$$\begin{array}{l} \frac{1}{2}\left(1-\sqrt{1-e^{-2D_{B}}}\right)\leq Error_{Bayes}\leq \frac{1}{2}e^{-D_{B}} \end{array}$$ (31)

This formula quantitatively binds the L5 Law of Distribution Overlap with the optimal error lower bound, serving as a core theoretical basis for task paradigm design.

Furthermore, the number of intention classes and the prior distribution also regulate the level of the Bayes error. As the number of intention classes C increases, the probability of overlap among classes in the observation space rises, and the Bayes error tends to increase overall. The more imbalanced the class priors, the lower the absolute value of the Bayes error; however, the corresponding chance-level accuracy also increases simultaneously, and the actually achievable performance improvement margin is correspondingly narrowed.

**6) Dynamic evolution of the Bayes error under non-stationary and closed-loop scenarios**

In static scenarios, the Bayes error is determined by a fixed observation distribution. However, in the dynamic closed-loop system of BCI, the Bayes error evolves continuously over time, forming a complete correspondence with the T4 Closed-Loop Convergence Theorem and the L7/L8 Laws.

When the neural representation undergoes distribution shift over time, the overlap of class-conditional distributions increases persistently, and the Bayes error grows monotonically with time, leading to a continuous decay in the performance of a statically trained decoder. The larger the shift amplitude and the stronger the non-stationarity, the faster the rate at which the Bayes error increases. This is the core essence of the performance degradation in long-term BCI system operation.

In contrast, under an effective feedback learning mechanism, the user optimizes task-relevant neural representations through neural plasticity, the separability of class distributions continues to improve, the overlap decreases, and the Bayes error monotonically declines with the training process, eventually converging to the minimum achievable Bayes error under the current observation conditions. The timeliness and accuracy of the feedback directly determine the rate of decline and the final convergence value of the Bayes error.

**7) Practical estimation of the Bayes error and performance tightening pathways**

In light of the unknown distributions and limited samples characteristic of real BCI scenarios, the Bayes error can be estimated or constrained through several methods, and its lower bound can be continuously tightened through engineering approaches. For practical estimation, a lower bound estimation method based on distribution distances can be adopted: by calculating metrics such as the Bhattacharyya distance and Fisher discriminant ratio from training samples, and substituting them into the corresponding formulas, a conservative lower bound for the Bayes error can be obtained. This method does not require estimating the complete probability distribution and is computationally simple and robust. Alternatively, an empirical upper bound approach using cross-validation can be employed: the error of the optimal classifier obtained through cross-validation serves as an empirical upper bound for the Bayes error; the better the classifier's performance and the stronger its generalization ability, the closer this upper bound is to the true Bayes error.

From an engineering optimization perspective, the Bayes error is not an unchangeable fixed value; it can be continuously tightened through full-chain optimization: improving the SNR of acquisition hardware, optimizing the spatial configuration of electrodes, designing highly discriminative task paradigms, introducing closed-loop feedback training, and adopting adaptive algorithms to compensate for distribution shift can all reduce the overlap of class distributions, essentially compressing the lower bound of the Bayes error and enhancing the optimal achievable performance of the system.

**8) Theoretical significance**

The mathematical foundation of this theorem rests on classical results from statistical decision theory. The core contribution of this paper lies not in redefining the Bayes error, but in anchoring the general theory of optimal error to the complete observation chain of BCI systems. It systematically dissects the regulatory mechanisms by which BCI-specific factors—including neural noise, electrode configuration, signal-to-noise ratio, class overlap, temporal drift, and closed-loop feedback—modulate the performance lower bound, and establishes a quantitative mapping from hardware parameters and paradigm design to the theoretical performance upper limit.

### 4.4 T4: BCI closed-loop convergence theorem

**1) Assumptions**

(1) The closed-loop system satisfies first-order Markov dynamics.
(2) Feedback induces dynamic evolution of the neural encoding distribution, i.e., $P_{t+1}\left(\;A|I\right)=I\left(P_{t}\left(\;A|I\right),F_{t}\right)$, where *F*_*t*_ denotes the feedback signal.
(3) Information transmission satisfies a finite-capacity constraint: *MI*(*I*_*t*_; *S*_*t*_) ≤ **C**
(4) At any time t, the decoding error is bounded by the Bayes error under the distribution at that time.
(5) Under the regulation of closed-loop feedback, the user's neural encoding strategy is updated in a direction that enhances the separability of task-related neural representations, such that the expectation of intention mutual information is non-decreasing over time, i.e., *E*[*MI*(*I*; *S*)_*t*+1_]≥*E*[*MI*(*I*; *S*)_*t*_ ].

2) **Theorem statement**

Closed-loop feedback can dynamically adjust the neural encoding and the observation channel, causing *MI*(*I*_*t*_; *S*_*t*_) to change over time, but it always satisfies *MI*(*I*_*t*_; *S*_*t*_) ≤ *C*, while also satisfying Equation 32.


$$\begin{array}{l} \underset{t\to \infty }{\lim}P\left(Î=I\right)\leq 1-Error_{Bayes}^{*} \end{array}$$ (32)

where *Error*_Bayes_^*^ is the minimum achievable Bayes error under the current observation channel.

**3) Proof**

By the effective learning condition in Assumption (5), the expectation of intention mutual information is non-decreasing over time, indicating that the expected amount of intention information contained in the observed signal increases continuously throughout the learning process. According to T1, an increase in mutual information implies an increase in the amount of information about the intention in the observed signal; according to T3, the Bayes error is constrained by the observation distribution. Therefore, closed-loop adaptation can improve the theoretically achievable information bound, but the actual improvement in classification performance also depends on the decoder structure, the task distribution, and changes in the decision boundary. By the T2 Observation Information Upper Bound Theorem, *MI*(*I*_*t*_; *S*_*t*_) ≤ *MI*(*I*; *A*) has an upper bound, so the mutual information cannot grow indefinitely and must converge to a certain limit value. Correspondingly, the limit of decoding accuracy is determined by the Bayes error under this limiting mutual information and cannot exceed the theoretically optimal value under the current observation conditions, i.e., the accuracy upper bound is *Error*_Bayes_^*^.

**4) Information-theoretic effects of non-stationarity**

Closed-loop feedback directly reshapes the information structure by altering the neural encoding distribution, and its dynamic evolution of mutual information satisfies Equation 33.


$$\begin{array}{l} \Delta MI_{t}=MI\left(I_{t+1};S_{t+1}\right)-MI\left(I_{t};S_{t}\right) \end{array}$$ (33)

Under favorable learning conditions, *E*[Δ*MI*_*t*_]≥0. The feedback-driven closed-loop adaptation process can continuously optimize the neural representation and decoding strategy, enabling the effective transmission of intention information through the brain–computer interaction channel to increase progressively and gradually approach the theoretical capacity upper bound of the observation channel.

However, non-stationarity has a dual impact on information estimation and transmission efficiency. On the one hand, through adaptive learning and neural plasticity regulation, the user can enhance the stability and separability of task-relevant neural representations, thereby increasing the effective mutual information between the observed signal and the intention variable (Saway et al., 2024). On the other hand, the distribution shift caused by changes in neural state, cognitive state, and the external environment can lead to time-varying biases in mutual information estimation based on fixed data distributions, and cause data distribution mismatch in statically trained decoding models, thereby reducing the efficiency of neural information utilization (Karpowicz et al., 2025). Therefore, a BCI system must establish a balance between dynamic adaptation mechanisms and information stability constraints to maintain effective information transmission capability during long-term interaction.

Consequently, information estimation for closed-loop BCIs must adopt time-varying estimation frameworks such as sliding windows and online updates to accurately reflect the system's true information state.

**5) Theoretical significance**

It should be emphasized that the convergence conclusion of this theorem strictly depends on the effective learning condition in Assumption (5), and it describes the convergence trend of closed-loop BCI systems under ideal conditions rather than an inevitable outcome for all closed-loop BCI systems. When feedback quality is insufficient, the update direction of neural representations deviates from the task goal, or significant non-stationary drift exists, mutual information may decrease or oscillate over time, and the system does not necessarily converge toward improved performance. The core value of this theorem lies in clarifying the necessary prerequisites for closed-loop convergence, indicating that the adaptive mechanisms of practical systems must be centered on ensuring effective feedback, controlling update step sizes, and suppressing non-stationary drift, so as to drive the system to satisfy the theorem's prerequisites and converge toward performance improvement. Meanwhile, this theorem also provides a theoretical explanation for the typical learning curve characteristic in BCI training—rapid improvement followed by plateau saturation—and forms a coherent theoretical correspondence with Law L7 (human–machine co-adaptation) and Law L8 (non-stationary dynamics).

### 4.5 Discussion on the tightness of the theoretical limits

The aforementioned theorems have provided, from the perspectives of information theory and statistical decision theory, the decodability, information transmission constraints, and optimal performance limits of BCI systems. However, it is necessary to further discuss under what conditions these theoretical bounds are “tight,” and why there is still a significant gap between real BCI systems and the theoretical limits. Analyzing these issues not only helps to clarify the applicable scope of the theoretical results, but also reveals the fundamental reasons for the limited performance of current BCI systems.

First, for the information upper bound in Theorem T2, *MI*(*I*; *S*) ≤ *MI*(*I*; *A*),, its tightness depends on the fidelity of the observation mapping *S* = *H*(*A*)+ε. When the observation process is nearly noise-free, the mapping *H*(·) is close to invertible, and the observation dimensionality is sufficiently high, the observed signal can retain the intention-related neural information relatively completely, and at this time *MI*(*I*; *S*)≈*MI*(*I*; *A*),, with the information upper bound nearly achieved. However, in real BCI systems, the observation is typically affected by factors such as multi-source aliasing, limited spatial sampling, volume conduction effects, and sensor noise, leading to irreversible loss of a large amount of neural information already at the observation stage (Brinkmann, 2024; Zhang and Ahmed, 2026). Moreover, if the observed signal contains non-neural confounding variables related to the intention, the actual decoding performance may appear “artificially high,” seemingly breaking through the theoretical upper bound, but in essence this is due to spurious correlations brought by confounding components rather than a genuine improvement in neural decoding capability. This is also one of the important reasons why some BCI experimental results are difficult to reproduce and generalize poorly across scenarios. Therefore, there are significant hierarchical differences in the tightness of this upper bound across different observation modalities: invasive spike and ECoG systems have high observation fidelity, so the upper bound is relatively tight; scalp EEG and fNIRS systems have strong volume conduction effects and large information loss, so the upper bound is looser and closer to a theoretically achievable limit.

Furthermore, for the Bayes error lower bound in Theorem T3, i.e., *Error*_*dec*_≥*Error*_*Bayes*_,, it can theoretically be achieved only when the decoder equals the optimal maximum a posteriori (MAP) classifier under the true posterior distribution. In other words, only when the system accurately obtains the true probability distribution *P*(*I*∣*S*) and has infinite training data and sufficient model capacity can the decoding error approach the Bayes limit. However, real BCI systems often cannot meet these conditions. On the one hand, brain signals are characterized by high noise, low signal-to-noise ratio, and significant inter-individual variability (Phillips et al., 2026); on the other hand, issues such as finite samples, model mismatch, training bias, and insufficient optimization all cause the actual decoder to deviate from the theoretically optimal strategy (Kim et al., 2024). Therefore, the Bayes error in practice should be regarded more as an “unbreakable theoretical lower bound” rather than a performance metric that can usually be strictly achieved.

Moreover, even when the mutual information is relatively high, the actual system performance may not necessarily approach the theoretical limit. This is because mutual information describes the “overall statistical dependence,” whereas actual decoding performance is also affected by factors such as the geometric structure of the distribution, the degree of class overlap, finite-sample effects, and model generalization capability. For example, when the conditional distributions corresponding to different intentions exhibit significant overlap, the system may still have a high irreducible error even if the mutual information is non-zero. Similarly, under conditions of limited training samples, the theoretically existing optimal decision boundary may not be accurately estimated, resulting in actual performance significantly lower than the theoretical optimum.

On the other hand, although the closed-loop feedback mechanism can improve the stability and separability of task-relevant features through neural plasticity (Williams et al., 2025), its performance improvement is also constrained by the theoretical bounds. According to T4, feedback can alter the neural representation distribution and enhance decodability, but it cannot break through the theoretical limit jointly determined by the amount of information and the Bayes error. Therefore, the performance saturation phenomenon after long-term training does not necessarily mean algorithm failure, but rather more likely reflects that the system has gradually approached the theoretical upper bound under the current observation conditions (Atilla et al., 2024).

In summary, the theoretical results given by T1–T4 more characterize the reachable bounds and unbreakable bounds of BCI systems, rather than the performance that real systems will necessarily achieve. It should be pointed out that BCI performance optimization depends not only on the decoding algorithm itself, but also on the synergistic improvement of multiple levels, including task design, observation methods, information structure, and feedback learning. The aforementioned theoretical limits are all based on the assumptions that the true distribution is known and the decoder achieves Bayes optimality. However, in real systems, the decoder can only be trained from finite samples, and is limited by model capacity, generalization error, inter-individual variability, and non-stationarity, causing the performance of the actual decoder to further deviate from the theoretical optimum. In low signal-to-noise ratio and small-sample scenarios, the learnability gap is often the most dominant source of the discrepancy between actual performance and the theoretical limits.

### 4.6 Key issues in information-theoretic analysis of BCI systems

Although information theory provides a unified mathematical tool for characterizing the encoding, transmission, and decoding processes of intention information in BCI systems, the estimation of mutual information, the characterization of information distribution, and channel capacity analysis still face many challenges due to the high dimensionality, low signal-to-noise ratio, non-stationarity, and multi-source mixing characteristics of neural signals. Therefore, information-theoretic analysis for BCI needs to focus not only on the amount of information itself, but also consider the influence of limited observation conditions, neural encoding mechanisms, and the closed-loop human–machine adaptation process on the efficiency of information transmission.

#### 4.6.1 Methods for estimating mutual information of neural signals and finite-sample bias

In BCI systems, the mutual information MI(I;S) between the intention variable and the neural observation signal is a core metric for measuring system decodability. However, because neural data typically exhibit high dimensionality and small sample size, mutual information is difficult to compute directly and accurately, and its estimation is inevitably affected by the number of samples, feature dimensionality, and signal-to-noise ratio.

Currently, commonly used mutual information estimation methods mainly include plug-in estimation and nearest-neighbor-based methods. The plug-in method estimates the probability distributions *p*(*I*), *p*(*S*), and the joint distribution *p*(*I, S*), then substitutes them into the definition of mutual information for calculation. Its advantage is intuitive form and simple implementation, but in high-dimensional neural signal spaces it is susceptible to probability density estimation errors and produces notable overestimation of mutual information under finite-sample conditions. In contrast, the k-nearest neighbor (k-NN) estimation method estimates information density through local distance relationships among samples, typically yielding lower estimation bias in high-dimensional, small-sample scenarios, and is thus widely used in neuroscientific and brain-computer interface research.

Nevertheless, even with non-parametric estimation methods, finite-sample bias is still unavoidable, and the degree of bias generally increases as the sample size decreases and the signal dimensionality increases. Therefore, it is necessary to establish a reliable information quantity benchmark by combining methods such as cross-validation, analytic bias correction, and permutation tests. In BCI applications, the effectiveness of mutual information estimation depends not only on the estimation method itself, but is also jointly influenced by the signal-to-noise ratio, sample size, and feature selection strategy. This is also an important factor limiting the transfer of information-theoretic metrics from theoretical analysis to practical system evaluation.

#### 4.6.2 Multi-scale distribution characteristics of information

According to the principle of low-dimensional neural representation (P3), intention-related information is not uniformly distributed throughout the entire neural signal space, but is concentrated in specific temporal, spatial, and frequency subspaces. Therefore, BCI information analysis needs to characterize the distribution regularities of effective information from a multi-scale perspective.

On the temporal scale, neural information exhibits distinct dynamic evolution characteristics. For instance, the information in evoked paradigms such as P300 and SSVEP is typically concentrated within a specific time window after the stimulus, whereas the event-related synchronization/desynchronization (ERS/ERD) information in the motor imagery paradigm gradually accumulates with the task duration and exhibits information saturation phenomena.

On the spatial scale, intention-related information is mainly concentrated in the electrodes or channels corresponding to task-relevant brain regions, while irrelevant channels contribute more as noise. Therefore, the essence of channel selection and spatial filtering is to search for observation dimensions with higher information contribution, thereby improving the effective signal-to-noise ratio.

On the frequency scale, different tasks depend on different neural oscillatory patterns. For example, motor imagery mainly involves event-related synchronization and desynchronization changes in the mu/beta bands, while some higher-order cognitive tasks may be accompanied by enhanced activity in the gamma band.

Therefore, the BCI feature extraction process is essentially a process of searching for and compressing information-rich spatio-temporal-frequency subspaces, with the goal of maximizing the retention of intention-related mutual information while reducing data dimensionality.

#### 4.6.3 Distortion effects of neural source aliasing on the information channel

In non-invasive BCIs, neural source aliasing caused by the volume conduction effect is a significant factor leading to information loss in the observed signal. Since signals such as scalp EEG are actually the mixed result of multiple neural source activities after spatial projection, unavoidable information compression exists between the observation space and the true neural source space.

From an information-theoretic perspective, neural source aliasing mainly causes two effects. First, high-dimensional neural source activities are mapped to a low-dimensional observation space, where information from different sources is mixed in the same observation channel, preventing some source information from being independently recovered, thereby causing irreversible information loss. Second, the aliasing process increases the degree of overlap between the conditional distributions of different intention classes, reduces class separability, and raises the lower bound of the Bayes optimal classification error.

Therefore, the theoretical role of spatial filtering methods (such as CSP, beamforming, etc.) is not simply to improve classification accuracy, but rather, under the constraints of the observation channel, to reduce the degree of source aliasing through approximate inverse mapping and increase the effective information retention rate, thereby enhancing the information transmission capacity of the BCI system.

#### 4.6.4 Task design and optimization of BCI system capacity

The information capacity of a BCI system is determined not only by signal acquisition and decoding algorithms, but also by upstream constraints from task design and neural encoding strategies. The selection of task parameters is essentially an optimization between information transmission efficiency and the user's cognitive cost.

First, there exists a capacity trade-off relationship regarding the number of classes. Increasing the number of control intention classes can raise the theoretical information content, but at the same time reduces the separability of neural representations among classes and increases the user's cognitive load, causing the actual growth of mutual information to saturate. Therefore, there exists an optimal class size for BCI systems that maximizes the information transfer rate.

Second, a similar trade-off exists for trial duration. Longer stimulation or task times can accumulate more neural information and increase single-trial mutual information, but reduce the control frequency per unit time. Thus, the system capacity depends on the balance between the information accumulation rate and the time cost.

Furthermore, different BCI paradigms possess different information encoding characteristics. Exogenous evoked paradigms such as SSVEP and P300 have high encoding stability and signal-to-noise ratio, and are suitable for high-speed communication tasks; endogenous paradigms such as motor imagery, although exhibiting larger inter-individual variability and lower signal-to-noise ratio, have the advantages of requiring no external stimulus and supporting autonomous control. The difference in information capacity between the two essentially reflects the differences in the characteristics of the neural encoding channel.

#### 4.6.5 Low-dimensional neural representations from the information bottleneck perspective

The principle of low-dimensional neural representation (P3) proposed earlier can be further explained through information bottleneck theory. The neural encoding process does not maximize the preservation of all neural activity information, but rather compresses irrelevant information while ensuring the transmission of intention-related information. This objective can be formally expressed as Equation 34.


$$\begin{array}{l} \min_{p\left(A|I\right)}H\left(A\right)-\beta \cdot MI\left(I;A\right) \end{array}$$ (34)

where β is a weighting coefficient for information retention. The optimal solution of this optimization is necessarily a low-dimensional, task-relevant representation, because high-dimensional redundant components increase representation complexity without contributing intention information.

This theory, from the perspective of the trade-off between information efficiency and metabolic cost, explains why the brain adopts low-dimensional distributed encoding to carry intention information, and also provides a theoretical basis at the neural mechanism level for low-dimensional feature modeling in BCI.

## 5 BCI fundamental laws

Building upon the BCI neuroscientific principles (P1–P4) in Section 3 and the BCI fundamental theorems (T1–T4) in Section 4, this section abstracts and unifies the intrinsic constraint relationships revealed therein, proposing a set of BCI fundamental laws, Laws 1–8 (L1–L8). The BCI fundamental laws referred to in this paper are system-level structural regularities within the domain of engineering science, rather than universal laws at the level of fundamental natural science. Their establishment is mathematically supported by rigorous theoretical theorems and empirically grounded in neuroscientific empirical regularities and BCI engineering experience, possessing verifiable explanatory, predictive, and constraining capabilities.

Unlike the theorems, which emphasize conclusions that are rigorously provable under specific model assumptions, these laws characterize the common constraints and regularities that hold stably in general BCI systems, reflecting the intrinsic limitations of BCI systems in the processes of information generation, transmission, and closed-loop regulation. These laws characterize the limiting factors of BCI decoding performance from the three levels of information theory, statistical structure, and dynamic systems, providing a theoretical basis for BCI system design and performance evaluation. It should be noted that this section is not a restatement of the theorems from Section 4; rather, on the basis of relaxing specific model assumptions, it further abstracts and refines the intrinsic constraint relationships revealed by them, forming system-level regularities with generality. At the same time, each law clearly specifies its derivation source, predictive capability, and applicable boundaries, distinguishing them from scattered empirical design intuitions.

### 5.1 L1: law of signal-to-noise ratio constraining decoding performance

The following quantitative relationships hold under the model assumption of an additive Gaussian noise linear observation channel.

**Law Statement:** The effective signal-to-noise ratio of a BCI system is composed of intrinsic neural noise, volume conduction aliasing noise, physiological artifact noise, and sensor noise, among others. Information loss caused by upstream noise cannot be recovered through downstream denoising, and there exists an incompressible noise floor determined by intrinsic neural noise. This composite signal-to-noise ratio constrains the amount of intention information in the observed signal as well as decoding performance, as expressed in Equations 35, 36.


$$\begin{array}{l} MI\left(I;S\right)=f\left(SNR\right),\;f^{\prime }\left(SNR\right)>0 \end{array}$$ (35)

$$\begin{array}{l} SNR=\frac{∥A_{task}{∥}^{2}}{∥\varepsilon {∥}^{2}} \end{array}$$ (36)

L1 indicates that decoding performance is not constrained by a single signal-to-noise ratio, but is systematically governed by a four-stage cascaded noise structure: sensor noise and physiological artifacts can be largely eliminated through hardware and algorithms, volume conduction aliasing noise can only be partially compensated by spatial filtering, whereas intrinsic neural noise—arising from stochastic neuronal firing and inter-trial variability—constitutes an incompressible performance ceiling. For a linear observation channel with additive Gaussian noise, the observation channel capacity and the SNR satisfy the corresponding relationship of Shannon's formula, as shown in Equation 37.


$$\begin{array}{l} C_{obs}=\frac{1}{2}\log_{2}\left(1+SNR\right) \end{array}$$ (37)

where the channel capacity *C*_*obs*_ is the maximum amount of neural information that the observation stage can carry, directly determining the upper bound of the intention mutual information. Combined with the cascaded channel constraint, the intention mutual information and the SNR satisfy a monotonically increasing concave function relationship, as shown in Equation 38.


$$\begin{array}{l} MI\left(I;S\right)\propto \log_{2}\left(1+SNR\right) \end{array}$$ (38)

This quantitative relationship indicates that the decoding performance gain brought by SNR improvement exhibits diminishing marginal returns: in the low SNR regime, improving the SNR can significantly increase the amount of effective information; when the SNR reaches a relatively high level, the performance benefit from further improvement will tend to level off. This regularity also explains why noise reduction and signal quality improvement are fundamental optimization approaches for BCI systems, yet a clear performance ceiling exists.

The rigorous mathematical derivation of this law originates from the T1 decodability theorem and the capacity formula of additive noise channels, yet it is not a mere special case of T1: T1 only establishes a quantitative relationship between mutual information and decoding performance at an abstract level, without addressing what physical factors constrain mutual information itself. L1 further decomposes the signal-to-noise ratio into four stages of noise sources with differing degrees of compensability, revealing hierarchical irreversibility and an incompressible noise floor, and provides a system-level explanation for cross-modal performance differences and denoising resource allocation that T1 cannot offer. The core predictive power of this law is manifested in two aspects: first, SNR and decoding performance follow a logarithmic growth relationship, with diminishing marginal returns in performance gain—denoising optimization yields far greater benefits in low-SNR scenarios than in high-SNR scenarios; second, the theoretical upper bound of decoding performance can be quantitatively estimated from the SNR level, providing a basis for judging the expected returns of system optimization. This law applies to all BCI modalities and task paradigms, but the dominant factors influencing SNR differ across modalities: the SNR of invasive spike-based BCIs is primarily affected by stochastic neuronal firing noise, whereas that of non-invasive BCIs such as scalp EEG is mainly determined by volume conduction effects and physiological artifacts.

### 5.2 L2: law of low-dimensional neural representation of intention

**Law Statement:** intention-related neural information is embedded in the form of a low-dimensional structure within the high-dimensional neural activity space, which can be represented by Equations 9, 10, i.e., *A* = *A*_*task*_+*A*_*noise*_, dim(*A*_*task*_) ≪ dim(*A*).

L2 indicates that the decodable information in BCI is mainly concentrated in a low-dimensional task-relevant subspace, while a large number of components in the high-dimensional space are irrelevant to decoding or constitute interference. This law can be rigorously interpreted through information bottleneck theory: the neural encoding process is essentially a metabolic efficiency trade-off between “preserving intention information” and “reducing representation complexity,” with its optimization objective shown in Equation 39. This relationship is grounded in the information bottleneck theoretical framework, independent of any specific form of class-conditional distributions, and is applicable to general probability distributions.


$$\begin{array}{l} \min_{p\left(A|I\right)}H\left(A\right)-\beta \cdot MI\left(I;A\right) \end{array}$$ (39)

where *H*(*A*) is the entropy of neural activity (representation complexity), and β > 0 is the weight for information retention. The optimal solution to this optimization problem is necessarily a low-dimensional manifold embedded in the high-dimensional neural space, i.e., retaining only low-dimensional information components related to the intention while discarding all redundant high-dimensional noise dimensions.

From an engineering perspective, this means that performing a low-dimensional projection on neural signals will not lose the effective intention-related mutual information; instead, it can reduce the estimation bias of mutual information under high-dimensional, small-sample conditions and improve the generalization capability of the decoding model.

This law is jointly supported by the P3 Principle of Low-Dimensional Neural Representation and information bottleneck theory, and can be rigorously derived from the metabolic efficiency optimization objective of neural encoding. Its core predictive capability is: the effective intention-related information is embedded only in a low-dimensional subspace, and excessively increasing the signal acquisition dimensionality and feature dimensionality will not increase the amount of effective decoding information, but will instead introduce the curse of dimensionality and the risk of overfitting. This law applies to the vast majority of BCI paradigms based on population neural activity; only in extreme scenarios such as high-precision single-neuron decoding will the low-dimensional representation property be somewhat weakened.

### 5.3 L3: law of irreversible observation information loss

**Law Statement:** The information loss in the observation process can be decomposed into two components of fundamentally different nature. The first is geometric irreversible loss, which arises from source aliasing caused by volume conduction and the compression of observational dimensionality; its magnitude is determined by the spatial resolution of observation and cannot be recovered by any decoding or signal processing algorithm. The second is noise-induced compensable loss, which is determined by the quality of the observed signal and can be partially compensated through hardware upgrades and digital denoising algorithms. Under this loss structure, the information retention rates of different observation modalities exhibit a stable hierarchical ordering, which is uniquely determined by the magnitude differences of geometric irreversible loss. The amount of information about intention satisfies Equation 20, i.e., *MI*(*I*; *S*) ≤ *MI*(*I*; *A*), and strictly *MI*(*I*; *S*) < *MI*(*I*; *A*) when noise or non-invertible mappings are present.

L3 indicates that the conversion of neural signals from central neural activity to observed signals inevitably incurs information loss, and this process is generally irreversible. In conjunction with the information difference relationship given by Theorem T2, the total information loss produced by observation can be decomposed into two independent types of loss: one is the source aliasing loss, caused by the superposition and mixing of multiple neural source signals due to volume conduction and dimension compression, which is a permanent, irrecoverable loss brought about by the geometric structure of the observation; the other is the noise loss, originating from sensor noise and various physiological artifacts, which can be partially compensated for through hardware upgrades and digital noise reduction algorithms. The information retention rate can be further defined as $\eta =\frac{MI\left(I;S\right)}{MI\left(I;A\right)}$, used to quantify the information fidelity capability of the observation system. The information retention rate of invasive BCIs is significantly higher than that of non-invasive BCIs, which is also the core information-theoretic reason for their superior decoding performance.

This law is mathematically grounded in the T2 observational information upper bound theorem (data processing inequality), yet it is not a mere restatement of T2: T2 only proves and provides a formal decomposition of loss at an abstract level, without explicitly distinguishing which portion of the loss is geometrically irreversible and which can be compensated by technical means, nor does it offer hierarchical ordering predictions for the information retention rates of different observation modalities. L3 further points out the algorithmic irrecoverability of source aliasing loss and establishes a hierarchical ordering of information retention rates—“Spikes > ECoG > scalp EEG > fNIRS”—providing engineering guidance for observation system selection and spatial resolution optimization that T2 cannot deliver.

This law is directly derived from the T2 Observation Information Upper Bound Theorem (data processing inequality) and is the core constraint regularity of the BCI observation chain. Its core predictive capability lies in: the information loss in the observation stage can be decomposed into source aliasing loss and noise loss; the former is determined by the spatial resolution of the observation and constitutes irreversible loss, while the latter is determined by signal quality and can be partially compensated for through technical means. This law applies to all BCI observation systems and serves as the core theoretical basis for explaining why the performance of invasive BCIs is generally superior to that of non-invasive BCIs.

### 5.4 L4: law of observation dimensionality constraint

**Law Statement:** The dimensionality of the observed signal is far lower than that of the true neural state space, as shown in Equation 8, i.e., dim(*S*) ≪ dim(*A*), and the observation mapping *S* = *H*(*A*) is a non-bijective mapping, thus making it impossible to uniquely recover the complete neural state.

L4 reveals the incompleteness of BCI observation from a geometric perspective: a high-dimensional neural manifold is projected into a low-dimensional observation space, resulting in information compression and loss. The constraint imposed by observational dimensionality on information capacity can be characterized by information-theoretic laws of high-dimensional signal sampling: for a linear mixing model with neural source-space dimensionality n and observational dimensionality m under additive Gaussian noise, the maximum information capacity of the observation channel satisfies Equation 40.


$$\begin{array}{l} C_{obs}\leq \min\left(m,n\right)\cdot \log_{2}\left(1+SNR\right) \end{array}$$ (40)

When *m* ≪ *n*, the observation dimension becomes the core bottleneck of information capacity. This formula also indicates that increasing the number of observation channels can linearly raise the theoretical upper bound of channel capacity, but limited by the source aliasing effect and the signal-to-noise ratio level, the growth of actual mutual information exhibits a diminishing marginal return.

This law is jointly derived from the P2 Principle of Multi-Source Aliasing Observation and the capacity constraint of the linear observation channel. Its core predictive capability is: there is a positive correlation with diminishing marginal returns between the number of observation channels and the information capacity; when the number of channels reaches a threshold, the performance gain from further increasing channels will be insufficient to offset the increase in hardware and computational cost. This law applies to all BCI modalities with spatially distributed acquisition, providing a theoretical basis for the design and selection of electrode arrays and channel counts.

### 5.5 L5: law of overlapping distributions of different intentions

**Law Statement:** The degree of distribution overlap among different intentions in the observation space is not a fixed value, but is systematically regulated by four factors: paradigm encoding mechanism, number of classes, time window length, and neural representation stability. Moreover, there exists a paradigm-dependent irreducible error floor. The distribution overlap of exogenous evoked paradigms is determined by the quantization of stimulus parameters, whereas that of endogenous paradigms is determined by individual differences in neural representations, and the irreducible error floor of the latter is systematically higher than that of the former. For any two classes of intention i and j, with conditional probability densities *p*(*S*|*I* = *i*) and *p*(*S*|*I* = *j*), respectively, the distribution overlap integral is defined as shown in Equation 41:


$$\begin{array}{l} OV\left(i,j\right)=\int_{S}\min\left\{p\left(S|I=i\right),p\left(S|I=j\right)\right\}dS \end{array}$$ (41)

If *OV*(*i, j*) > 0, then an irreducible error exists, i.e., *Error*_*Bayes*_ > 0.

The degree of class distribution overlap bears a strict quantitative relationship with mutual information and the Bayes error. For the two-class intention scenario, the Bhattacharyya distance *D*_*B*_ is defined to measure the overlap between the two conditional distributions, as shown in Equation 42.


$$\begin{array}{l} D_{B}=-\ln\left(\int \sqrt{p\left(S|I=i_{1}\right)p\left(S|I=i_{2}\right)}ds\right) \end{array}$$ (42)

The higher the degree of distribution overlap, the smaller *D*_*B*_, and the corresponding mutual information and Bayes error satisfy Equation 43.


$$\begin{array}{l} MI\left(I;S\right)\leq 1-e^{-D_{B}},\;Error_{Bayes}\leq \frac{1}{2}e^{-D_{B}} \end{array}$$ (43)

That is, the degree of distribution overlap directly determines the upper bound of mutual information and the lower bound of decoding error. When the two distributions completely overlap, *D*_*B*_ = 0, the mutual information is 0, and the Bayes error equals the chance level, forming a complete correspondence with the T1 Decodability Theorem.

This law is mathematically grounded in the T3 Bayes error lower bound theorem, yet it is not a mere restatement of T3. T3 establishes a static lower bound on the Bayes error given the observational distribution and quantifies the association between distribution overlap and error via the Bhattacharyya distance; L5 further points out that distribution overlap itself is not fixed, but is systematically regulated by upstream factors such as paradigm encoding mechanism, number of classes, and neural representation stability, and different paradigms exhibit different levels of irreducible error floor. This dynamic regulatory law at the paradigm level is not covered by 3′s static distribution analysis, and its mathematical foundation lies in the correspondence between distribution separability and error in statistical decision theory.

The core predictive power of this law is that the degree of class distribution overlap determines the irreducible error lower bound of decoding, which cannot be overcome solely through algorithmic optimization; reducing distribution overlap through task design is the fundamental path to substantially improving decoding performance. This law applies to all classification-based BCI tasks, whereas regression-based tasks correspond to the irreducible distortion lower bound under rate-distortion theory.

### 5.6 L6: law of intention separability constraining decoding performance

**Law Statement:** Decoding performance is constrained by the separability of the class-conditional distributions in the observation space, as shown in Equation 44.


$$\begin{array}{l} P\left(Î=I\right)\leq g\left(separability\left(P\left(S|I\right)\right)\right) \end{array}$$ (44)

where *g*(·) is a monotonic function that maps separability to the performance upper bound; *separability*(·) is a metric that measures how well-separated the distributions of different classes are (not unique in form).

L6 characterizes the constraint of the static statistical distribution structure on decoding performance: the upper limit of decoding performance is determined by the degree of distinguishability between the observation distributions corresponding to different intentions.

Class separability is an intermediate variable connecting the geometric structure of the distribution with the information-theoretic bounds. Taking the Fisher discriminant ratio *J* as a quantitative separability metric, under the assumption of two-class Gaussian distributions with equal covariance, it satisfies a monotonically positive correlation with mutual information and the upper bound of classification accuracy, as shown in Equation 45.


$$\begin{array}{l} MI\left(I;S\right)=g\left(J\right),\;g^{\prime }\left(\cdot \right)>0 \end{array}$$ (45)

This quantitative relationship indicates that all methods for improving class separability (such as feature engineering, discriminative models, contrastive learning) essentially increase effective mutual information by enlarging the degree of separation of class distributions in the observation space, thereby raising the theoretical upper limit of decoding performance.

This law is jointly supported by the T1 and T3 Theorems and statistical discriminant analysis theory, and is a core regularity connecting the geometric structure of distributions with decoding performance. Its core predictive capability is: the upper limit of decoding performance is determined by class separability, and the benefit of all algorithm optimizations cannot exceed the theoretical upper limit corresponding to separability; the core role of discriminative models is to fully exploit the separability potential, rather than to create additional room for performance improvement. This law applies to all supervised decoding BCI paradigms and provides a theoretical reference for evaluating the effectiveness of algorithm optimization.

### 5.7 L7: law of human–machine co-adaptation

**Law Statement:** Under the closed-loop feedback mechanism, the neural representation and the decoding model co-evolve, as shown in Equation 3, i.e., *P*(*A*_*t*_∣*I*_*t*_) → *P*(*A*_*t*+1_∣*I*_*t*+ 1_).

The human–machine co-adaptation process manifests at the information-theoretic level as a dynamic gain in intention mutual information. Let the intention mutual information at time t be *MI*_*t*_(*I*; *S*), and the information gain brought about by feedback regulation be Δ_*t*_; then the information dynamics of co-adaptation is given by Equation 46.


$$\begin{array}{l} MI_{t+1}\left(I;S\right)=MI_{t}\left(I;S\right)+\Delta_{t} \end{array}$$ (46)

Under effective learning conditions, *E*[Δ_*t*_]≥0, meaning that mutual information is non-decreasing with the interaction process. The co-adaptation process has a convergence boundary: when *MI*_*t*_(*I*; *S*) approaches the observation channel capacity *C*_*obs*_, the information gain Δ_*t*_ tends toward 0, and the system performance saturates.

This regularity explains the learning curve of BCI training from an information-theoretic perspective: performance improves rapidly in the initial phase and then gradually enters a plateau phase, the essence of which is that the effective mutual information progressively approaches the physical limit of the observation system.

### 5.8 L8: law of non-stationary dynamics

**Law Statement:** The statistical properties of a BCI system change dynamically over time, as shown in Equation 4, i.e., *P*(*S*_*t*_∣*I*_*t*_)≠*P*(*S*_*t*+1_∣*I*_*t*+ 1_).

L8 indicates that due to the combined effects of feedback, learning, and environmental changes, the neural representation and its observation distribution exhibit non-stationarity. At the information-theoretic level, non-stationarity manifests as a time-varying drift of the conditional distribution *p*(*S*|*I*), and its core impact on the decoding system is a decrease in the utilization rate of effective mutual information. Let the distribution during the training phase be *p*_*train*_(*S*|*I*) and the distribution during the actual operation phase be *p*_*test*_(*S*|*I*); the distribution difference between them can be quantified by the KL divergence *D*_*KL*_(*p*_*test*_∥*p*_*train*_ ).

The larger the distribution drift, the lower the utilization rate of the true mutual information by a decoder trained on the training distribution, and the larger the gap between actual decoding performance and the theoretical optimal performance. In this case, the effective information of a static decoder satisfies Equation 47.


$$\begin{array}{l} MI_{effective}\leq MI\left(I;S\right)-\alpha \cdot D_{KL}\left(p_{test}∥p_{train}\right) \end{array}$$ (47)

where α > 0 is the drift influence coefficient, characterizing the proportion of effective information attenuation caused by distribution drift. It should be noted that Equation 47 is a phenomenological or conceptual relationship; the specific value of α depends on the drift type, decoder structure, and data characteristics, and cannot be directly analytically derived from general information-theoretic principles, but must be estimated through experiments or simulations. This quantitative relationship provides a clear information-theoretic objective for adaptive decoding and domain adaptation algorithms: to maximize the utilization rate of the system's effective mutual information by compensating for distribution drift.

This law originates from the P4 Principle of Neural Plasticity and the time-varying characteristics of closed-loop systems, and is a core regularity distinguishing BCI systems from static pattern recognition systems. Its core predictive capability is that the performance of a statically trained decoder will naturally decay over time, with the decay rate positively correlated with the degree of neural distribution drift; adaptive algorithms can partially compensate for the drift, but cannot completely eliminate the influence of non-stationarity. This law applies to all long-term operating BCI systems and serves as the core constraint basis for system stability design.

The eight fundamental laws described above together constitute a complete system of constraints for BCI systems, which is fundamentally different from scattered engineering experience and design intuition: experience is fragmented qualitative cognition that can only indicate that a certain factor is important; laws, on the other hand, are systematic, quantitative regularities within a unified theoretical framework. They can not only explain common phenomena in the BCI field, but also quantitatively predict the performance bounds and evolutionary trends of a system, providing verifiable theoretical constraints for full-chain system design. Elevating from empirical intuition to systematic laws can provide a unified set of regularities for BCI research to move from empirical trial-and-error toward being theory-driven.

## 6 Theoretical case study of a typical SSVEP-BCI paradigm

To verify the operability and practical explanatory power of the above theoretical framework in a concrete BCI paradigm, this section selects a typical four-class steady-state visual evoked potential (SSVEP) BCI as a theoretical case study. It should be emphasized that this case study does not constitute a new independent experiment; rather, under a physiologically meaningful parametric observation model, it maps the scientific hypotheses, neuroscientific principles, fundamental theorems, and basic laws proposed in Sections 2–5 onto a concrete BCI task, demonstrating how theoretical results can be translated into computable system performance bounds.

### 6.1 Paradigm and parametric modeling

Consider a four-class SSVEP-BCI system. The system comprises four visual stimulus targets, each encoded with a periodic flicker frequency of 6, 7, 8, and 9 Hz, respectively, corresponding to four discrete control intentions. The user generates a corresponding SSVEP neural response by fixating on one of the targets, and the system identifies the user's intention based on task-related features in the EEG associated with the stimulus frequencies and their harmonics.

Let the user's control intention at time t be *I*_*t*_∈{1, 2, 3, 4}, and assume a uniform prior distribution over the four classes, as shown in Equation 48.


$$\begin{array}{l} P\left(I_{t}=i\right)=\frac{1}{4},\;i=1,2,3,4 \end{array}$$ (48)

Accordingly, the prior entropy of the four-class task is given by Equation 49.


$$\begin{array}{l} H\left(I_{t}\right)=-\overset{4}{\underset{i=1}{\sum }}P\left(I_{t}=i\right)\log_{2}P\left(I_{t}=i\right)=2\;bit \end{array}$$ (49)

In the absence of any brain signal information, the random-guessing accuracy is $P_{chance}=\frac{1}{4}=\;25\%$.

The acquisition system employs three occipital EEG channels—Oz, O1, and O2—with a sampling rate of 250 Hz and a single-trial duration of 4 s. To construct a theoretical scenario under finite-sample conditions, each target class is assumed to comprise 60 trials, yielding a total of 240 trials. It should be emphasized that these parameters serve only to establish a theoretical analysis scenario, and the subsequent theoretical bounds do not depend on any specific experimental dataset.

Following the unified formalization model in Section 2.1, define the latent neural activity corresponding to the user's intention as *A*_*t*_, the EEG observation signal as *S*_*t*_, and the decoder output as ${\hat{I}}_{t}$; the system can be represented as ${\hat{I}}_{t}$. The neural encoding process is further expressed as shown in Equation 50.


$$\begin{array}{l} A_{t}|I_{t}=i\sim N\left(\mu_{i},\Sigma_{A}\right) \end{array}$$ (50)

and the EEG observation process is approximated as *S*_*t*_ = *HA*_*t*_+*N*_*t*_. where *H*∈ℝ^3 × *n*^ is the observation mapping matrix from the latent neural activity space to the three EEG channels, n is the dimensionality of the latent neural activity, and *N*_*t*_ is the observation noise. To remain consistent with the paper's analysis of information loss due to limited observation and volume conduction, *n*≫3 is assumed, and the observation noise is approximated as Gaussian.

The effective observation SNR is set to 3 dB, representing a scalp EEG observation scenario under moderate noise conditions. The SNR parameter here is used only to characterize the SNR constraint discussed in L1, and is not directly converted into single-trial intention mutual information; the subsequent *MI*(*I*_*t*_; *S*_*t*_) is derived separately using a parametric effective observation model.

From the perspective of neuroscientific principles, this SSVEP paradigm directly corresponds to P1–P3 of this paper. SSVEP responses originate from the coordinated activity of multiple neural subpopulations in the primary visual cortex and associated visual areas, consistent with the distributed neural encoding principle described in P1; the three-channel scalp EEG constitutes a low-dimensional aliased observation of high-dimensional neural source activity projected through volume conduction, with observational dimensionality m = 3 far lower than the latent neural source dimensionality n and a non-bijective observation mapping, corresponding to the multi-source aliasing and incomplete observation principle in P2 and the observational dimensionality constraint in L4; task-related information is concentrated in the narrowband subspaces of the stimulus frequencies and their second and third harmonics, with an effective intrinsic dimensionality far lower than that of the raw signal, consistent with the low-dimensional task-related neural representation principle in P3 and L2. The above correspondences indicate that the parametric modeling of this case study is not an arbitrary setup, but a reasonable abstraction of a typical SSVEP system under the constraints of the principle layer of this paper.

### 6.2 Decodability assessment and SNR constraint

According to T1, a necessary condition for the statistical decodability of the user's intention in the observation space is *MI*(*I*_*t*_; *S*_*t*_) > 0. For this four-class SSVEP case study, to obtain an analytical result, a four-state task-related observation variable *Y*_*t*_ = *g*(*S*_*t*_) is formed by extracting features from the EEG associated with the stimulus frequencies and their harmonics. Assume that, given the true intention, the probability that this observation correctly corresponds to the true class is *P*, while the remaining three classes are confused with equal probability, as shown in Equation 51.


$$P\left(Y_{t}=j|I_{t}=i\right)=\{\begin{array}{c} p,\text{\&}j=i,\\ \frac{1-p}{3},\text{\&}j\neq i.\; \end{array}$$ (51)

This model corresponds to a four-class symmetric observation channel. To construct a baseline case with *MI*(*I*_*t*_; *Y*_*t*_)≈1.25 *bit*, *p* = 0.8731 is adopted. Since the four-class priors are uniform and the observation channel is symmetric,


$$\begin{array}{l} H\left(Y_{t}\right)=2\;bit \end{array}$$

and


$$\begin{array}{l} H\left(Y_{t}|I_{t}\right)\\ \;=-p\log_{2}p-\left(1-p\right)\log_{2}\frac{1-p}{3}\\ \;\approx 0.750\;bit\\ \; \end{array}$$

Therefore,


$$\begin{array}{l} MI\left(I_{t};Y_{t}\right)=H\left(Y_{t}\right)-H\left(Y_{t}|I_{t}\right)\approx 2-0.750=1.25\;bit \end{array}$$

Since *Y*_*t*_ is a function of the EEG observation *S*_*t*_, by the data processing inequality *MI*(*I*_*t*_; *S*_*t*_)≥*MI*(*I*_*t*_; *Y*_*t*_), it follows that


$$\begin{array}{l} MI\left(I_{t};S_{t}\right)\geq 1.25\;bit>0 \end{array}$$

Therefore, this four-class SSVEP paradigm satisfies the statistical decodability condition of T1, i.e., the EEG observation contains non-zero information about the user's intention, and there exists a statistical decoder with performance superior to the random-guessing baseline.

Furthermore, applying Fano's inequality to the four-class uniform prior,


$$\begin{array}{l} H\left(I_{t}|S_{t}\right)\leq h_{2}\left(P_{e}\right)+P_{e}\log_{2}3 \end{array}$$

where


$$\begin{array}{l} H\left(I_{t}|S_{t}\right)=H\left(I_{t}\right)-I\left(I_{t};S_{t}\right) \end{array}$$

Under the parametric scenario with *MI*(*I*_*t*_; *S*_*t*_)≈1.25*bit*,


$$\begin{array}{l} H\left(I_{t}|S_{t}\right)\approx 2-1.25=0.75\;bit \end{array}$$

Therefore, the error rate satisfies


$$\begin{array}{l} 0.75\leq h_{2}\left(P_{e}\right)+P_{e}\log_{2}3 \end{array}$$

Numerical solution yields


$$\begin{array}{l} P_{e}≳0.127 \end{array}$$

i.e.,


$$\begin{array}{l} Acc_{\max}^{Fano}≲87.3\% \end{array}$$

The 87.3% here is a Fano-type conservative performance bound given by mutual information, rather than a guaranteed accuracy of any specific classification algorithm. This result is clearly above the 25% random-guessing level for a four-class task, consistent with the insight of T1 that “non-zero intention information implies the existence of a decoder superior to the random baseline.”

From the perspective of L1, the SNR of 3 dB in the baseline case indicates that the observed signal is still significantly constrained by noise and source aliasing. For an ideal real-valued Gaussian observation channel, the theoretical capacity per observation vector can be written as


$$\begin{array}{l} C=\frac{m}{2}\log_{2}\left(1+SNR\right) \end{array}$$

where *m* = 3; when *SNR* = 3 *dB*, i.e., a linear SNR of 2, we have


$$\begin{array}{l} C=\frac{3}{2}\log_{2}\left(3\right)\approx 2.38\;bit \end{array}$$

This value is the capacity upper bound under ideal independent channels; actual EEG channels exhibit spatial correlation due to volume conduction, so the effective capacity will be lower than this value. This result serves only to illustrate the SNR constraint revealed by L1, and is not equivalent to the single-trial intention mutual information. The effective intention information in practical BCI systems is further jointly constrained by factors such as the task-related subspace, observational aliasing, and the statistical decoding structure.

### 6.3 Observational information upper bound and information loss

Under the four-class uniform prior condition, the maximum information content of the user's intention is *H*(*I*_*t*_) = 2 *bit*. To construct an ideal neural encoding baseline, assume that the four classes of SSVEP intention possess fully separable task-related representations in the latent neural activity space; then


$$\begin{array}{l} MI\left(I_{t};A_{t}\right)=H\left(I_{t}\right)=2\;bit \end{array}$$

Here, *MI*(*I*_*t*_; *A*_*t*_)=2 bits is the ideal neural encoding baseline, i.e., it assumes no encoding loss for the four-class intention at the neural activity level, with the entire intention entropy converted into neural mutual information. This baseline serves only to quantify the information loss at the observation stage and does not represent the actual information capacity of real neural encoding.

On the other hand, from the parametric observation model in Section 6.2,


$$\begin{array}{l} MI\left(I_{t};S_{t}\right)\approx 1.25\;bit \end{array}$$

According to the data processing inequality in T2,


$$\begin{array}{l} MI\left(I_{t};S_{t}\right)\leq MI\left(I_{t};A_{t}\right) \end{array}$$

Therefore, the observational information loss in this case study is


$$\begin{array}{l} \Delta I_{obs}=MI\left(I_{t};A_{t}\right)-MI\left(I_{t};S_{t}\right)\approx 0.75\;bit \end{array}$$

and the corresponding information retention rate is


$$\begin{array}{l} \frac{MI\left(I_{t};S_{t}\right)}{MI\left(I_{t};A_{t}\right)}=\frac{1.25}{2}=62.5\% \end{array}$$

That is, under this parametric scenario, approximately 37.5% of the intention-related information from the ideal neural encoding level to the actual EEG observation level is not retained by the current observation system.

According to L3 and L4, the information loss at the observation stage can be understood from two aspects: on the one hand, the limited number of channels and volume conduction cause high-dimensional neural activity to be projected into a low-dimensional observation space, forming irreversible source aliasing and geometric compression—this dimensionality constraint is precisely the observational dimensionality bottleneck characterized by L4; on the other hand, sensor noise and physiological artifacts further degrade the effective signal quality. The former constitutes information loss caused by the observation structure itself, whereas the latter leaves some room for engineering compensation. Therefore, improving channel quality, optimizing spatial acquisition, and reducing noise can enhance the information retention rate, but cannot in general restore a finite observation to the complete neural state.

This result is consistent with the theoretical conclusion of T2: the observation process can only retain or lose intention information already present in neural activity, and cannot create new neural information out of nothing. Accordingly, further improvement of SSVEP system performance cannot rely solely on decoding algorithms, but must also address observation quality and signal fidelity.

### 6.4 Bayes error bound and class distribution overlap

T3 shows that, given *P*(*S*_*t*_∣*I*_*t*_), the classification error of any decoder cannot fall below the Bayes error. L5 further points out that the distribution overlap of different intentions in the observation space is an important source of irreducible error.

To illustrate this relationship, consider the most difficult-to-distinguish pair of adjacent stimulus frequencies, e.g., 7 and 8 Hz. Assume that the task-related EEG features of the two classes follow Gaussian distributions with equal covariance:


$$\begin{array}{l} S_{t}|I_{t}=i\sim N\left(\mu_{i},\Sigma \right) \end{array}$$

then the Mahalanobis distance between the two classes is given by Equation 52.


$$\begin{array}{l} d_{M}^{2}={\left(\mu_{1}-\mu_{2}\right)}^{T}\Sigma^{-1}\left(\mu_{1}-\mu_{2}\right) \end{array}$$ (52)

Under an illustrative parameter setting, *d*_*M*_≈1.8 is adopted. When the two conditional distributions have equal covariance, the Bhattacharyya distance can be written as $D_{B}=\frac{1}{8}d_{M}^{2},$; therefore,


$$\begin{array}{l} D_{B}\approx \frac{1}{8}\times 1.8^{2}\approx 0.405 \end{array}$$

According to the binary classification error bound given by the Bhattacharyya distance,


$$\begin{array}{l} P_{e}^{\left(2\right)}\geq \frac{1}{4}\left(1-\sqrt{1-e^{-2D_{B}}}\right) \end{array}$$

Substituting *D*_*B*_≈0.405 yields


$$\begin{array}{l} P_{e}^{\left(2\right)}≳12.7\% \end{array}$$

This result indicates that there exists irreducible statistical overlap for the 7 Hz vs. 8 Hz class pair, which is the most difficult to distinguish. It should be particularly noted that the above result is a theoretical lower bound for this binary class pair, and cannot be directly regarded as the overall Bayes error of the four-class SSVEP system; the overall four-class performance requires simultaneous consideration of the joint conditional distributions of all four classes and their prior probabilities.

From the perspective of L6, the above Mahalanobis distance and Bhattacharyya distance are essentially quantitative measures of class separability. Taking the Fisher discriminant ratio as an example, for two-class Gaussian distributions with equal covariance, the Fisher ratio $J=d_{M}^{2}$, which exhibits a monotonically positive correlation with mutual information and the upper bound of classification accuracy. In this case study, *d*_*M*_≈1.8 corresponds to *J*≈3.24, indicating moderate separability between the 7 and 8 Hz representations; if the frequency interval is increased to raise *d*_*M*_ to 2.5, then *J*≈6.25, with significantly enhanced separability and a correspondingly elevated accuracy upper bound. This is consistent with the insight of L6 that the upper limit of decoding performance is determined by class separability.

From the paradigm design perspective of L5, the above relationship indicates that enhancing the separability of neural representations evoked by different stimulus targets can reduce the overlap of class conditional distributions, thereby lowering the irreducible error. If the interval between stimulus frequencies is increased and the corresponding mean difference in task-related features is enlarged, then $d_{M}^{2}\;$increases accordingly, further leading to an increase in *D*_*B*_ and a decrease in the corresponding error lower bound.

Therefore, the theoretical significance of L5 lies in revealing that SSVEP decoding performance is not determined solely by the classification algorithm; the class separability formed by the task paradigm itself constitutes a more fundamental performance constraint. By optimizing stimulus frequency configuration, task classes, and other paradigm parameters, class distribution overlap can be reduced at the source of information, providing greater theoretical performance headroom for subsequent decoding algorithms.

### 6.5 Closed-loop and dynamic extensions

The calculations in Sections 6.2–6.4 above are based on a static distribution assumption, corresponding to theoretical bounds in an open-loop scenario. In the long-term use of practical SSVEP-BCIs, the system exhibits closed-loop dynamic characteristics, corresponding to P4, T4, L7, and L8 of this paper.

From the perspective of human–machine co-adaptation (L7), through continuous training the user can enhance the stability of neural responses to specific frequency stimuli, leading to a gradual improvement in the separability of task-related neural representations. Let the intention mutual information at the initial time be *MI*(*I*; *S*)_0_≈1.25 *bit*; under effective feedback training, the mutual information grows non-decreasingly with the training process, i.e., *MI*(*I*; *S*)_*t*+1_≥*MI*(*I*; *S*)_*t*_. According to T4, this growth process is constrained by the upper bound of the observation channel capacity; when the mutual information approaches the channel capacity, the gain tends to zero and the system enters a performance plateau. This dynamic process explains the common learning curve characteristic in SSVEP training—“rapid initial improvement followed by late-stage saturation.”

From the perspective of non-stationary dynamics (L8), fatigue, attention fluctuations, and changes in electrode impedance during prolonged use can cause distribution drift in neural representations, leading to performance degradation of a static decoder trained on the initial distribution. Let the KL divergence between the training distribution and the runtime distribution be *D*_*KL*_; the greater the drift, the lower the utilization of effective mutual information by the static decoder, and the larger the gap between actual performance and the theoretical upper bound. In such cases, adaptive decoding or domain adaptation methods are required to compensate for the drift, but the effects of non-stationarity cannot be completely eliminated, consistent with the predictions of L8.

The above closed-loop analysis demonstrates that the theoretical framework of this paper is applicable not only to performance bound computation in static open-loop scenarios, but also to characterizing the dynamic evolution of SSVEP systems during long-term interaction.

### 6.6 Summary of the case study

Using a four-class SSVEP-BCI as the vehicle, this case study parametrically instantiates the fundamental theorems (T1–T4), neuroscientific principles (P1–P4), and basic laws (L1–L8) within the theoretical framework of this paper, demonstrating a complete computational chain from paradigm parameters to neural encoding, observational information, decodability, Bayes performance bounds, and ultimately closed-loop dynamic evolution.

At the decodability level, the parametric observation model yields an intention mutual information of approximately 1.25 bits, from which a conservative accuracy upper bound of approximately 87.3% is obtained via Fano's inequality—significantly higher than the 25% random-guessing baseline—verifying the decodability assessment of T1. At the observational information level, using the ideal neural encoding as the baseline, the information retention rate at the EEG observation level is approximately 62.5%, translating the abstract conclusion of T2 regarding the observational information upper bound into a quantifiable information loss. At the class separability level, the Gaussian distribution overlap analysis of the adjacent 7 and 8 Hz classes yields a binary classification error lower bound of approximately 12.7%, illustrating from the perspective of L5 that optimizing stimulus frequency configuration can expand the system's theoretical performance headroom at the source.

It should be emphasized that the purpose of this case study is not to provide an empirical accuracy under a specific experimental condition, but to demonstrate how the theoretical chain proposed in this paper can be translated into *a priori* computable performance constraints in a concrete BCI paradigm. The above Fano-type accuracy upper bound of 87.3% is of the same order as the typical performance (75%−85%) of three-channel, four-class, short-time-window SSVEP reported in the public literature; the gap by which actual performance falls slightly below the theoretical upper bound can be explained by finite-sample effects, model mismatch, and individual differences, consistent with the tightness discussion in Section 4.5. This instantiation process demonstrates that the unified theoretical framework proposed in this paper can explain the performance bounds of SSVEP-BCI from three levels—intention information, observation quality, and class separability—and provides a concrete theoretical foundation for subsequent closed-loop adaptation and system design analysis.

## 7 Theory-guided design principles of BCI systems

The proposed BCI theoretical framework consists of multiple hierarchical layers, as illustrated in Figure 1, each serving distinct theoretical functions. The scientific hypotheses (H1–H4) and neuroscientific principles (P1–P4) characterize the biological foundations and underlying mechanisms that enable BCI systems. The fundamental theorems (T1–T4) further establish the decodability of intentions and the theoretical performance limits of such systems. Building upon these results, the fundamental laws (L1–L8) provide a higher level of abstraction, summarizing and generalizing the theoretical constraints into general principles that are applicable to practical system design.

Based on this hierarchical structure, this section focuses on translating the abstract theoretical constraints defined by L1–L8 into actionable engineering design principles. These principles can guide not only the development of signal processing and decoding algorithms, but also the design of BCI paradigms, including cognitive task design, external stimulus design, and human–machine interaction strategies.

Furthermore, beyond conventional performance evaluation metrics, this section also investigates the implications of L1–L8 for system stability and robustness under dynamic environments and long-term operation conditions. In particular, stability and robustness are analyzed in terms of time-varying statistical properties and sensitivity to distributional shifts, as characterized by the non-stationary nature of BCI systems.

### 7.1 Design principles based on L1: enhancing signal-to-noise ratio

Based on the SNR constraint revealed by L1, BCI system design should prioritize increasing the relative strength of task-relevant neural signals over noise in order to maximize the amount of usable information in the observed signal and thereby improve decoding performance.

From a paradigm design perspective, this can be achieved by enhancing task-evoked neural activations through the optimization of stimulus intensity, task duration, and trial repetition schemes. From an implementation perspective, signal quality can be further improved through high-information channel selection strategies, such as selecting electrodes over task-relevant brain regions, as well as through noise suppression techniques, including band-pass filtering, artifact removal, and independent component analysis (ICA). In addition, improvements in data acquisition hardware, including electrode quality and system stability, constitute another critical factor for SNR enhancement.

For performance evaluation, signal quality can be quantified using the signal-to-noise ratio defined as $SNR=\frac{∥A_{task}{∥}^{2}}{∥\varepsilon {∥}^{2}}$ and further assessed in conjunction with mutual information *MI*(*I*; *S*), providing an information-theoretic characterization of signal quality in BCI systems.

### 7.2 Design principles based on L2: low-dimensional neural representation modeling

According to L2, which characterizes the low-dimensional structure of intention-related neural representations, BCI systems should explicitly model or extract low-dimensional task-relevant neural subspaces to improve decoding efficiency and robustness.

From a paradigm design perspective, tasks should be designed to avoid excessive cognitive complexity or highly entangled mental processes, and instead favor paradigms that induce stable and consistent neural representations. From a representation learning perspective, low-dimensional embeddings can be obtained using techniques such as principal component analysis (PCA), common spatial patterns (CSP), and manifold learning methods, as well as deep learning approaches such as autoencoders that learn latent representations. In addition, task-related feature extraction methods such as event-related desynchronization/synchronization (ERD/ERS) can further enhance the discriminability of low-dimensional representations.

The effectiveness of low-dimensional modeling can be evaluated by estimating the intrinsic dimensionality of the data, analyzing class separability within the learned subspace, and comparing classification performance before and after dimensionality reduction.

### 7.3 Design principles based on L3 and L4: optimization of observation and signal acquisition

Based on the observational constraints and information preservation limits revealed by L3 and L4, BCI system design should focus on improving the efficiency and fidelity of intention-related information acquisition, rather than attempting to reconstruct the full neural state from observed signals.

From a paradigm design perspective, this can be achieved by selecting task paradigms that elicit stable and measurable neural activity patterns, and by optimizing stimulus design, task structure, and data acquisition strategies to minimize information loss. From an engineering perspective, multi-modal data fusion (e.g., combining EEG with fNIRS or fMRI) can be employed to increase information coverage across complementary measurement modalities. In addition, spatial filtering techniques such as beamforming can be used to enhance spatial resolution, while task-specific acquisition protocols can be designed to better align with the targeted neural dynamics.

At the system evaluation level, the effectiveness of observation systems can be assessed by analyzing the relationship between observation dimensionality, channel contribution, information retention ratio, and overall BCI performance.

### 7.4 Design principles based on L5: reduction of distributional overlap

According to L5, which establishes the relationship between class-conditional distribution overlap and irreducible decoding error, BCI system design should aim to minimize the overlap between different intention-related distributions in the observation space, thereby reducing unavoidable classification errors.

From a paradigm design perspective, this can be achieved by designing cognitive tasks with higher discriminative power, optimizing external stimulus configurations, and redefining or restructuring task categories to enhance the separability of neural representations associated with different intentions. From a modeling perspective, discriminative feature learning methods can be employed to improve class separation in the representation space.

The degree of separability can be quantitatively evaluated using measures such as distributional overlap *Overlap*(*P*(*S*∣*I*)), inter-class distance metrics, and divergence measures such as the Bhattacharyya distance.

### 7.5 Design principles based on L6: enhancing separability

Based on the separability constraint emphasized by L6, BCI system design should focus on enhancing the separability of different intentions in the observation space. At the level of BCI paradigm design, tasks or stimulus conditions capable of eliciting more pronounced neural differences can be preferentially selected to increase the distance between the neural representations of different intentions. At the level of model implementation, it is necessary to match the model complexity with the sample size to avoid overfitting under small-sample conditions. For scenarios with limited samples, low-complexity linear models, regularization constraints, or few-shot learning methods should be prioritized to improve the practical learnability of the decoder under limited data. In terms of evaluation, the effectiveness of separability enhancement can be comprehensively measured through metrics such as classification margin, Fisher discriminant ratio, classification accuracy, and AUC.

### 7.6 Design principles based on L7: human–machine co-adaptation

According to L7, which characterizes the mechanism of human–machine co-adaptation, BCI systems should be designed as dynamically co-evolving systems rather than static decoding models.

Accordingly, dynamic training strategies and real-time feedback mechanisms should be adopted to enable mutual adaptation between the user and the system during interaction. From an implementation perspective, online learning methods can be introduced to continuously update model parameters, while neurofeedback mechanisms can be incorporated to facilitate adaptive modulation of neural representations. In addition, individualized modeling strategies should be developed to account for inter-subject variability and ensure system adaptability across users.

The co-adaptive process can be quantitatively evaluated using learning curves, system convergence rate, and user adaptation metrics, allowing systematic characterization of human–machine interaction dynamics.

### 7.7 Design principles based on L8: handling non-stationarity

Based on the non-stationary dynamics revealed by L8, BCI systems must explicitly model or compensate for temporal variations in neural signals to ensure long-term stability of system performance.

Accordingly, cross-temporal adaptive task designs or dynamic updating strategies can be employed to mitigate the impact of time-varying statistical properties of neural activity. From an implementation perspective, adaptive filtering methods can be used for real-time signal adjustment, transfer learning techniques can enable cross-session knowledge transfer, and domain adaptation methods can be applied to reduce the impact of distribution shifts on decoding performance.

In terms of evaluation, non-stationarity can be characterized by detecting distribution shifts, i.e., *P*(*S*_*t*_∣*I*_*t*_)≠*P*(*S*_*t*+1_∣*I*_*t*+1_) together with cross-temporal performance degradation rates and robustness metrics, providing a quantitative assessment of the effectiveness of non-stationarity handling strategies.

The design criteria proposed in Sections 7.1–7.7 demonstrate that the optimization of BCI system performance does not rely solely on the complexity of the decoding algorithm, but is jointly determined by multiple factors, including paradigm design, signal quality, low-dimensional structural properties, class-distribution separability, and closed-loop adaptation mechanisms. These factors interact with one another, forming a constraint framework for BCI system design and evaluation, and providing theoretical guidance for realizing BCI systems with high performance and high stability.

## 8 Conclusion

This paper aims to provide a theoretical foundation for the analysis, design, and optimization of BCI systems. To this end, a BCI theoretical framework is constructed from the perspectives of information theory and statistical decision theory. First, the BCI system is formalized, and its core scientific questions and scientific hypotheses are clarified. On this basis, the neuroscientific principles underpinning the theoretical framework are further presented, BCI fundamental theorems and their proof ideas are proposed, and the corresponding fundamental laws are summarized.

The performance of a BCI system is not determined solely by the complexity of the decoding algorithm, but is fundamentally constrained by the amount of information contained in the observed signals, the information loss during the observation process, the separability of class distributions, and the human–machine co-adaptation dynamics under closed-loop interaction. These constraints constitute the theoretical basis for the performance boundaries of BCI systems, determining the relationship between their achievable capabilities and actual performance.

Building on this foundation, this study further establishes a mapping from the fundamental laws to system design, providing theoretical guidance for signal acquisition, feature construction, model design, and system optimization. The proposed framework establishes a clear connection among neuroscientific mechanisms, information-theoretic constraints, and engineering implementation, thereby facilitating the transition of BCI research from an empirically driven paradigm toward a theory-driven paradigm.

To verify the operability of the theoretical framework, this paper takes a four-class SSVEP-BCI as a case study, parametrically instantiates the theorems and laws one by one, and provides computable performance constraints including intention mutual information, Fano-type accuracy upper bound, observational information retention rate, and Bayes error lower bound for adjacent classes, which are compared against the typical performance magnitudes reported in the public literature. Building on this, the paper further establishes a mapping from the eight basic laws to system design guidelines, providing theoretical guidance for signal acquisition, feature construction, model design, paradigm construction, and closed-loop optimization. This framework establishes clear connections among neuroscientific mechanisms, information-theoretic constraints, and engineering implementation, and helps advance BCI research from empirical trial-and-error toward theory-driven practice.

The theoretical framework constructed in this paper still needs to be further validated and refined by integrating multimodal brain signals, different BCI paradigms, and long-term closed-loop experiments, so as to examine its applicability and generalization capability across different scenarios and conditions. This paper focuses on presenting the theorem derivations and the construction of the theoretical system under the core model. Future work will further extend the theoretical proofs to more complex scenarios such as non-stationary, multimodal, and continuous control settings, and conduct systematic validation and refinement of the theoretical framework by incorporating experimental data from multiple paradigms.

## Data Availability

No datasets were generated or analyzed during the current study. All relevant information and theoretical analyses are included in the article and/or supplementary material.
